# Landscape of pathogenic mutations in premature ovarian insufficiency

**DOI:** 10.1038/s41591-022-02194-3

**Published:** 2023-02-02

**Authors:** Hanni Ke, Shuyan Tang, Ting Guo, Dong Hou, Xue Jiao, Shan Li, Wei Luo, Bingying Xu, Shidou Zhao, Guangyu Li, Xiaoxi Zhang, Shuhua Xu, Lingbo Wang, Yanhua Wu, Jiucun Wang, Feng Zhang, Yingying Qin, Li Jin, Zi-Jiang Chen

**Affiliations:** 1 Center for Reproductive Medicine, Cheeloo College of Medicine, Shandong University, Jinan, China; 2 Key Laboratory of Reproductive Endocrinology of Ministry of Education, National Research Center for Assisted Reproductive Technology and Reproductive Genetics, Shandong Key Laboratory of Reproductive Medicine, Shandong Provincial Clinical Research Center for Reproductive Health, Jinan, China; 3 Obstetrics and Gynecology Hospital, Institute of Reproduction and Development, Fudan University, Shanghai, China; 4 School of Life Science and Technology, ShanghaiTech University, Shanghai, China; 5 State Key Laboratory of Genetic Engineering, School of Life Sciences, Human Phenome Institute, Zhangjiang Fudan International Innovation Center, Fudan University, Shanghai, China; 6 Shanghai Key Laboratory of Female Reproductive Endocrine Related Diseases, Shanghai, China; 7 Shanghai Key Laboratory for Assisted Reproduction and Reproductive Genetics, Shanghai, China; 8 Center for Reproductive Medicine, Ren Ji Hospital, School of Medicine, Shanghai Jiao Tong University, Shanghai, China; 9 Research Unit of Dissecting the Population Genetics and Developing New Technologies for Treatment and Prevention of Skin Phenotypes and Dermatological Diseases (2019RU058), Chinese Academy of Medical Sciences, Shanghai, China

**Keywords:** Disease genetics, Endocrine reproductive disorders

## Abstract

Premature ovarian insufficiency (POI) is a major cause of female infertility due to early loss of ovarian function. POI is a heterogeneous condition, and its molecular etiology is unclear. To identify genetic variants associated with POI, here we performed whole-exome sequencing in a cohort of 1,030 patients with POI. We detected 195 pathogenic/likely pathogenic variants in 59 known POI-causative genes, accounting for 193 (18.7%) cases. Association analyses comparing the POI cohort with a control cohort of 5,000 individuals without POI identified 20 further POI-associated genes with a significantly higher burden of loss-of-function variants. Functional annotations of these novel 20 genes indicated their involvement in ovarian development and function, including gonadogenesis (*LGR4* and *PRDM1*), meiosis (*CPEB1*, *KASH5*, *MCMDC2*, *MEIOSIN*, *NUP43*, *RFWD3*, *SHOC1*, *SLX4* and *STRA8*) and folliculogenesis and ovulation (*ALOX12*, *BMP6*, *H1-8*, *HMMR*, *HSD17B1*, *MST1R*, *PPM1B*, *ZAR1* and *ZP3*). Cumulatively, pathogenic and likely pathogenic variants in known POI-causative and novel POI-associated genes contributed to 242 (23.5%) cases. Further genotype–phenotype correlation analyses indicated that genetic contribution was higher in cases with primary amenorrhea compared to that in cases with secondary amenorrhea. This study expands understanding of the genetic landscape underlying POI and presents insights that have the potential to improve the utility of diagnostic genetic screenings.

## Abstract (web summary)

Whole-exome sequencing analyses in a cohort of 1,030 patients with premature ovarian insufficiency identify new likely pathogenic variants and reveal distinct genetic architectures between primary and secondary amenorrhea.

## Main

Premature ovarian insufficiency (POI), characterized by cessation of ovarian function^1,2^, affects 3.7% of women before the age of 40 years^3^ and remains a common cause of female infertility. The etiologies of POI are highly heterogeneous, and it can be caused by spontaneous genetic defects or induced by autoimmune diseases, infections or iatrogenic factors^4^. However, a large proportion of cases with POI are idiopathic, with multiple lines of evidence supporting a genetic basis for pathogenesis^5^. Identifying the molecular basis of POI is, thus, of paramount importance for investigating therapeutic targets, such as in vitro activation, and for guiding genetic counseling or pregnancy planning.

Recent advances in high-throughput sequencing have greatly expanded understanding of the pathogenesis of POI, with approximately 90 genes now linked to either isolated or syndromic POI^5–7^. However, variants in these known genes account for only a small fraction of patients, indicating the high genetic heterogeneity of POI^8^. Furthermore, limited sample sizes and inadequate controls in previous studies have prevented establishment of statistically robust single-gene associations and identification of novel causative genes. In this study, we performed, to our knowledge, the largest-scale whole-exome sequencing (WES) study in patients with POI to date and conducted a case–control analysis to systematically explore the genetic landscape of POI.

## Results

### Patient cohort

**Fig. 1:**
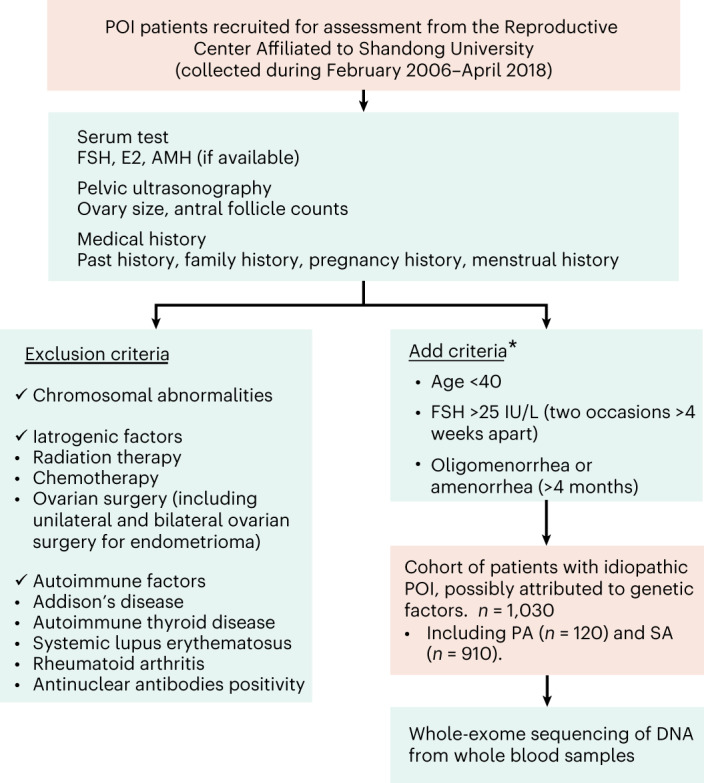
Flow chart for selecting the idiopathic POI cohort potentially attributable to genetic defects. A total of 1,790 patients with POI were recruited for the initial assessment, during which serum tests, pelvic ultrasounds and medical records were assessed for each participant. WES was performed in 1,030 patients who met inclusion criteria. *The inclusion criteria were based on 2016 ESHRE guidelines for POI. E2, estrogen.

We recruited 1,790 unrelated patients with POI from the Reproductive Hospital Affiliated to Shandong University for initial evaluation. Diagnosis of POI was based on the European Society of Human Reproduction and Embryology (ESHRE) guidelines: (1) oligomenorrhea or amenorrhea for at least 4 months before 40 years of age and (2) an elevated follicle stimulating hormone (FSH) level >25 IU L^−1^ on two occasions >4 weeks apart. Patients with chromosomal abnormalities and other known non-genetic causes of POI (including autoimmune diseases, ovarian surgery, chemotherapy and radiotherapy) were excluded. The final cohort included 1,030 unrelated patients with POI, consisting of 120 cases with primary amenorrhea (PA) and 910 cases with secondary amenorrhea (SA) (Fig. 1). The clinical characteristics are summarized in Supplementary Table 1. Among patients with SA, the mean age at the onset of oligomenorrhea or amenorrhea was 22.2 years.

In total, DNA extraction and WES was performed for 1,030 cases. Variant calling and annotation were conducted as described in Methods. Multiple sequence quality parameters were used to remove artifacts, and common variants (minor allele frequency (MAF) > 0.01 either in public controls from the gnomAD database^9^ or in-house controls from the HuaBiao project^10^) were filtered out (Methods).

### Identification of pathogenic variants in known POI-causative genes

**Extended Data Fig. 1:**
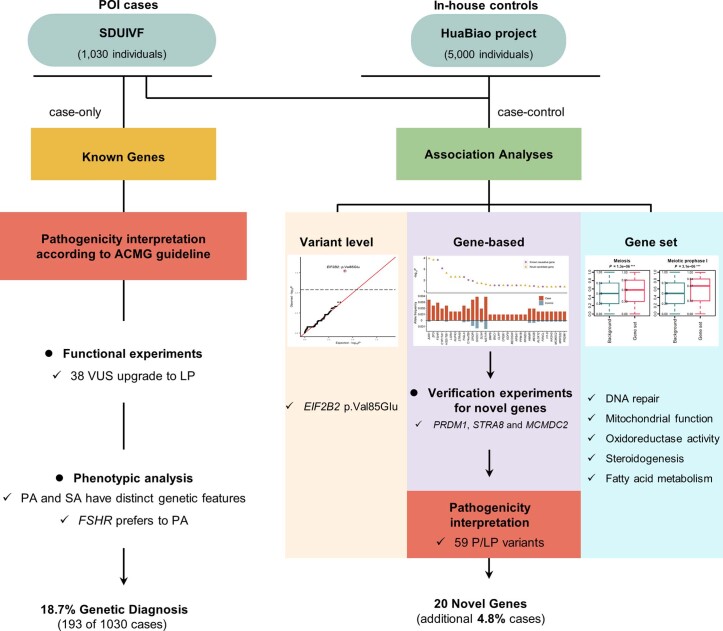
Graphical abstract. Summary of the WES analysis approach and findings. Abbreviations: SDUIVF, Hospital for Reproductive Medicine Affiliated to Shandong University.

**Extended Data Fig. 2:**
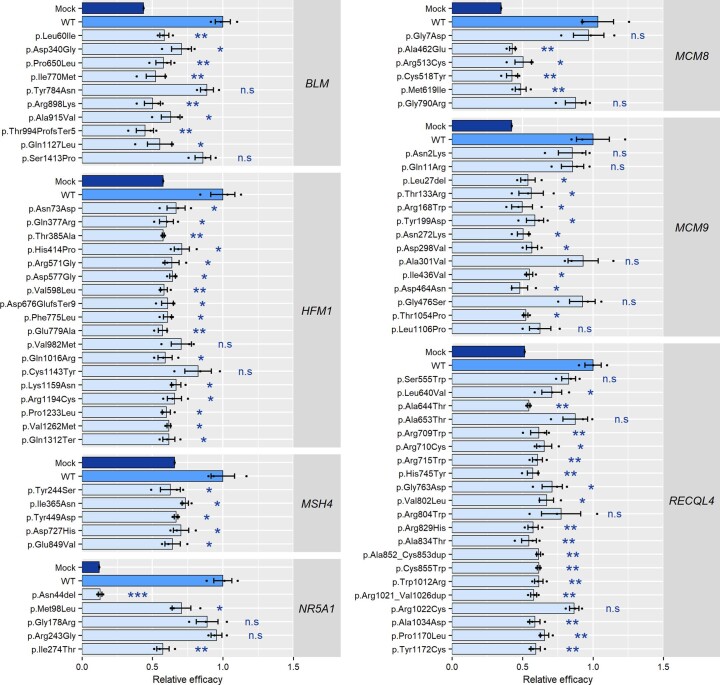
Experiment validation of variants with uncertain significance in seven genes. Variants with uncertain significance (VUS) identified in *BLM*, *HFM1*, *MCM8*, *MCM9*, *MSH4*, and *RECQL4* were verified through homologous recombination (HR) reporter system. Variants identified in *NR5A1* were verified through luciferase assay. The relative HR repair efficiency or transcriptional activity of wild-type (WT, blue column) and mutant (light blue column) proteins were compared with the mock group (cells transfected with pcDNA3.1 or pENTER vector, dark blue column) using two-sided Student’s t-test. Three independent experiments were conducted. Error bars indicate s.e.m. The numbers indicate *P* values. *P-value < 0.05. **P-value<0.01. ***P-value<0.001.n.s, not statistically significant. Source data

**Extended Data Fig. 3:**
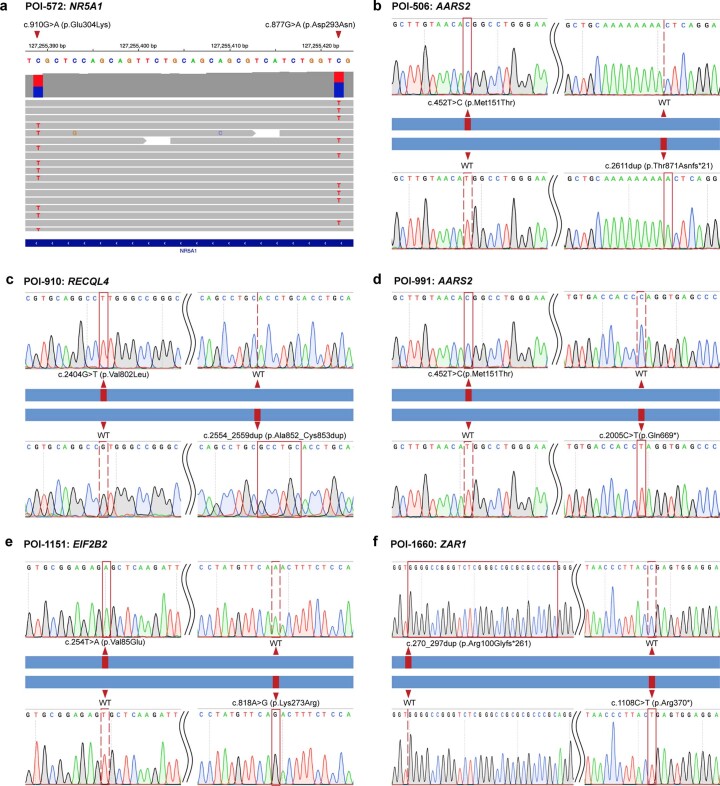
Phasing results of two P/LP variants in the same patient via IGV visualization and TA cloning sequencing. **a**, IGV visualization of mapped reads containing c.877 G > A and c.910 G > A detected in *NR5A1* in case POI-572. The two P/LP variants located in different reads and were confirmed to be in trans. **b**, TA clone sequencing results of c.452 T > C and c.2611dup detected in *AARS2* in case POI-506. The two P/LP variants located in different clones and were confirmed to be in trans. **c**, TA cloning sequencing results of c.2404 G > T and c.2554_2559dup detected in *RECQL4* in case POI-910. The two P/LP variants located in different clones and were confirmed to be in trans. **d**, TA cloning sequencing results of c.452 T > C and c.2005C > T detected in *AARS2* in case POI-991. The two P/LP variants located in different clones and were confirmed to be in trans. **e**, TA cloning sequencing results of c.254 T > A and c.818 A > G detected in *EIF2B2* in case POI-1151. The two P/LP variants located in different clones and were confirmed to be in trans. **f**, TA cloning sequencing results of c.270_297dup and c.1108 C > T detected in *ZAR1* in case POI-1660. The two P/LP variants located in different clones and were confirmed to be *in trans*.

**Extended Data Fig. 4:**
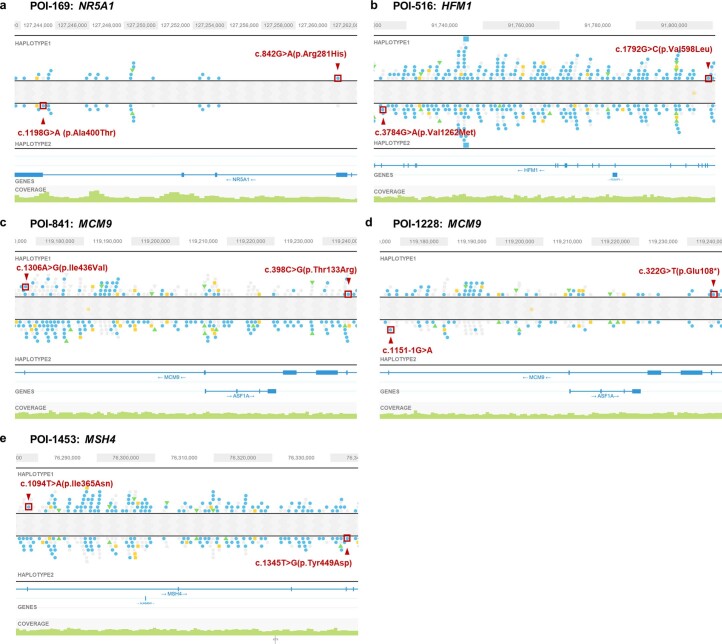
Phasing results of two P/LP variants in ultra long distance via 10×Genomics. **a**, Haplotype browser of c.842 G > A and c.1198 G > A detected in *NR5A1* in case POI-169. HAPLOTYPE 1 and HAPLOTYPE 2 are displayed as two separate tracks for multiple variations. The small icons represent SNPs (solid blue circles), small insertions (solid green triangles) and deletions (solid yellow rectangles). The GENES track displays annotated reference genes and the direction of each gene is indicated with arrows. Each vertical green bar in the COVERAGE track shows the average coverage-per-base for the area of the genome under the bar. The two variants were phased to different haplotype tracks and confirmed to be in trans. **b**, Haplotype browser of c.1792G > C and c.3784 G > A detected in *HFM1* in case POI-516. The two variants were phased to different haplotype tracks and confirmed to be in trans. **c**, Haplotype browser of c.398 C > G and c.1306 A > G detected in *MCM9* in case POI-841. The two variants were phased to the same haplotype track and confirmed to be in cis. **d**, Haplotype browser of c.322 G > T and c.1151-1 G > A detected in *MCM9* in case POI-1228. The two variants were phased to different haplotype tracks and confirmed to be in trans. **e**, Haplotype browser of c.1094 T > A and c.1345 T > G detected in *MSH4* in case POI-1453. The two variants were phased to different haplotype tracks and confirmed to be *in trans*.

**Extended Data Fig. 5:**
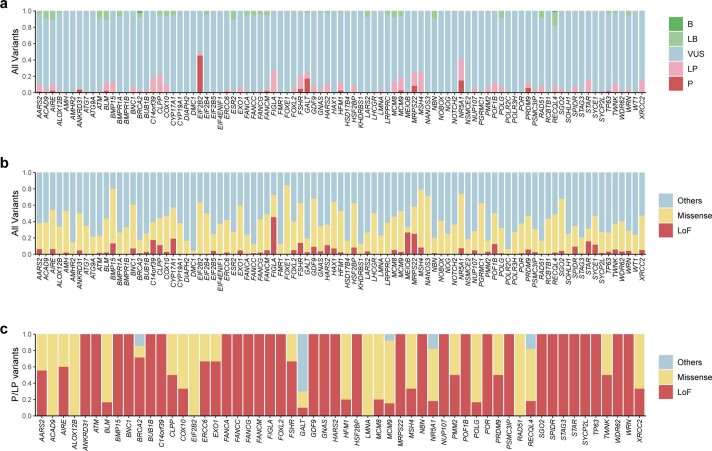
Overview of mutations identified in known POI genes. **a**, Distribution of pathogenic (P), likely pathogenic (LP), uncertain significance (VUS), likely benign (LB) and benign (B) variants across genes among all rare variants detected in known POI genes. **b**, Distribution of LoF, missense and other types (including in-frame indels and splice region) of all detected variants across genes. **c**, Distribution of LoF, missense and other types of P/LP variants across genes.

We first quantified the contribution yield (defined as the percentage of cases) to POI attributable to pathogenic variants in 95 well-characterized POI-causative genes (Supplementary Table 2). Variant pathogenicity in these known causative genes was evaluated by manual review following guidelines of the American College of Medical Genetics and Genomics (ACMG)^11^ or by ClinVar (Methods and Supplementary Table 3). Pathogenic (P) and likely pathogenic (LP) variants were prioritized for contribution analysis (Extended Data Fig. 1). Because variants of uncertain significance (VUSs) were likely to be upgraded to P/LP by introducing PS3 evidence via functional studies, we experimentally validated 75 VUSs from seven common POI-causal genes involved in homologous recombination (HR) repair (*BLM*, *HFM1*, *MCM8*, *MCM9*, *MSH4* and *RECQL4*) and folliculogenesis (*NR5A1*). Fifty-five variants were confirmed to be deleterious (Extended Data Fig. 2), among which 38 were upgraded to LP from VUS. The two P/LP heterozygous mutations occurring in the same gene in the same individual were confirmed to be in *trans* via T-clone or 10x Genomics approaches (Extended Data Figs. 3 and 4). The combined data, including the distribution of 4,730 variants detected in known genes, are shown in Extended Data Fig. 5.

**Fig. 2:**
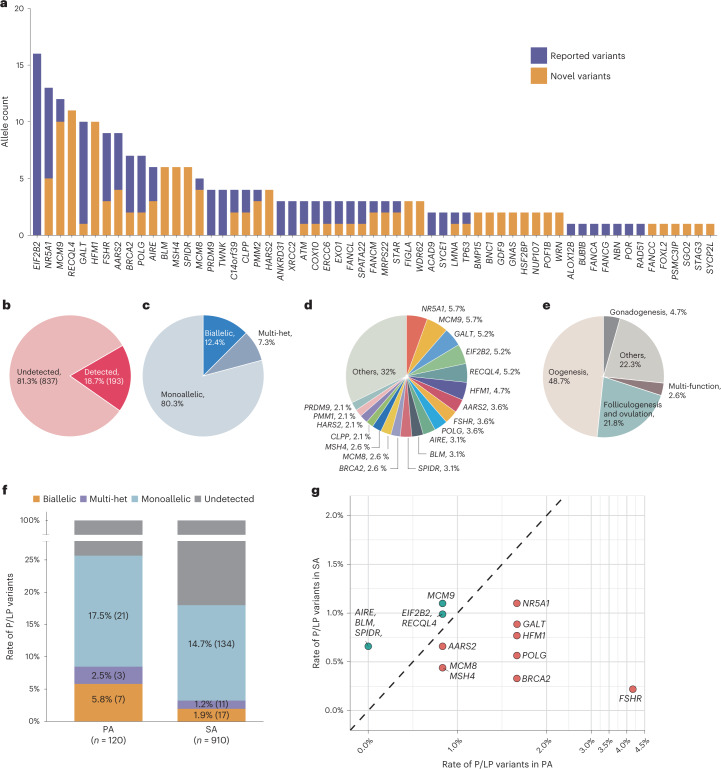
Overview of P/LP variants identified in known POI genes. **a**, Allele counts of P/LP variants detected in 59 of 95 known POI genes, including both novel and reported variants. Previously reported variants are those identified to be damaging according to ClinVar or published studies. **b**, Contribution yield of known POI-causative genes in 1,030 patients. **c**, The proportion of each mode of inheritance in the 193 patients carrying P/LP variants in known POI genes. **d**, The proportional contribution of each gene among 193 cases. **e**, The proportion of patients classified according to annotated function of the affected genes. ‘Oogenesis’ indicates genes involved in meiotic prophase I and HR. ‘Others’ indicates genes involved in the regulation of energy, metabolism and autoimmunity. ‘Multi-function’ refers to mutations in genes implicated in multiple pathways. **f**, The contribution rate of mode of inheritance in patients with PA and SA. **g**, The prevalences of P/LP variants in cases with PA and SA are shown for 15 genes detected in more than five cases.

Ultimately, 195 P/LP variants were identified across 59 known genes (Fig. 2), including 108 (55.4%) loss-of-function (LoF) variants, 81 (41.5%) missense, four (2.1%) inframe deletions or insertions and two (1.0%) splice regions. Specifically, LoF variants consisted of 38 frameshift deletions or insertions, 44 nonsense, 23 canonical splice site and three start–loss. Most P/LP variants (119, 61.0%), spanning 45 genes, were previously undocumented (Fig. 2a), including 76 LoF variants and 38 missense or inframe variants functionally verified in the present study (Extended Data Fig. 2). Among the 195 variants, 184 (94.4%) had PHRED-scaled CADD scores^12^ of greater than 20; nine (4.6%) with scores between 10 and 20; and just two with scores lower than 10. CADD is reasonably accurate in predicting the pathogenicity of variations, with both >10 and >20 having a similar predictive value. Among those genes, *EIF2B2* had the highest prevalence of pathogenic alleles in cases (16, 0.8%), due to the most recurrent variant p.Val85Glu (four heterozygotes, five homozygotes and one in *trans* with another LP variant p.Lys273Arg), which was described to cause SA in a Japanese patient due to compromised GDP/GTP exchange activity^13^.

The P/LP variants in known POI genes were detected in 193 patients, yielding an 18.7% contribution to POI incidence (Fig. 2b), among which most (155/193, 80.3%) carried monoallelic—that is, single heterozygous—P/LP variants, whereas 24 (12.4%) were identified with biallelic variants, and 14 (7.3%) had multiple P/LP variants in different genes (multi-het) (Fig. 2c). *NR5A1* and *MCM9*, respectively, had the highest prevalence in patients with genetic findings (11/193, 5.7%) (Fig. 2d) and emerged as the most frequently mutated genes in all patients (11/1030, 1.1%). Intriguingly, genes implicated in meiosis or HR accounted for the largest proportion (94/193, 48.7%) of detected cases, including *HFM1*, *SPIDR* and *BRCA2* (Fig. 2e). Genes responsible for mitochondrial function (*AARS2*, *ACAD9, CLPP*, *COX10*, *HARS2, MRPS22, PMM2, POLG* and *TWNK*) and metabolic (*GALT*) and autoimmune (*AIRE*) regulation also comprised a sizable proportion of known enriched genes, and these genes collectively accounted for 22.3% (43/193) of the detected cases (Fig. 2e). Although these genes have previously been linked with syndromic POI, our findings suggested the likelihood that impairment of pleiotropic genes might induce isolated POI.

### The distinct genetic characteristics of PA and SA

To explore the genetic features of different types of amenorrhea, we next compared the contribution yield of P/LP variants between patients with PA and SA. Among 120 patients with PA, 31 (25.8%) women carried P/LP variants, among whom 21 (17.5%) had monoallelic variants, seven (5.8%) had biallelic and three (2.5%) had multi-het (Fig. 2f). In comparison, patients with SA had a substantially lower overall contribution of P/LP variants (162/910, 17.8%), with 134 monoallelic (14.7%), 17 biallelic (1.9%) and 11 multi-het (1.2%). A considerably higher frequency of biallelic and multi-het P/LP variants was observed in patients with PA than with SA, indicating that the cumulative effects of genetic defects may affect clinical severity of POI.

To validate potential associations between genotype and phenotype, we determined the contributions of each causative gene to PA and SA. The results showed that *FSHR* was most prominently involved in PA (4.2% in PA versus 0.2% in SA), whereas putative pathogenic variants in *AIRE*, *BLM* and *SPIDR* were observed only in patients with SA in our cohort (none in PA versus 0.7% in SA) (Fig. 2g). Among them, *SPIDR* was previously reported in only a consanguineous family with PA^14^. The other 11 genes were not linked to a specific type of amenorrhea, including mutations in three genes (*HFM1*, *MSH4* and *POLG*) previously reported in SA and eight genes described in both PA and SA (Supplementary Table 4). These findings extended the phenotypic spectrum of known POI-causative genes.

### Association studies identified 20 novel POI candidate genes

To further investigate enriched genes and potential genetic defects associated with POI, we performed case–control association analyses at the variant and gene levels against in-house controls. The in-house control cohort was obtained from the HuaBiao project^10^, including 5,000 unrelated individuals, generated using the same exome capture kit as the cases and which had similar sequencing statistics (Methods and Supplementary Table 5).

**Extended Data Fig. 6:**
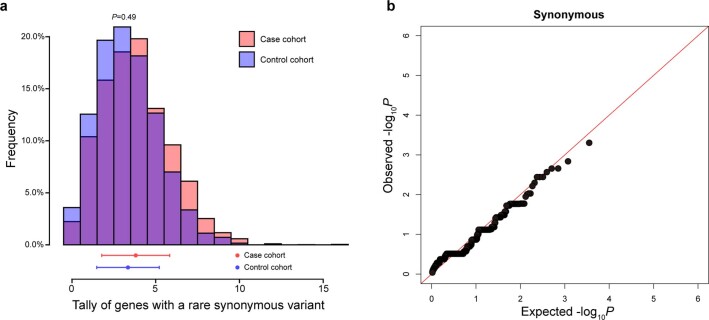
Association analyses of synonymous variants. **a**, Rates of gene with rare synonymous qualifying variants in 703 POI candidate genes. The upper panel shows the distribution of tally of genes carrying at least a rare synonymous qualifying variant among all tested genes when comparing the POI case cohort (red distribution, n = 1,030) to the control cohort (blue distribution, n = 5,000). We found no statistically significant difference between case and control contribution (two-sided Wilcoxon rank sum test, *P* = 0.49). The lower panel shows mean (points) and SD (bars) of qualifying genes of the case cohort (red) and the control cohort (blue). **b**, The quantile-quantile plot of the expected versus observed P values comparing the burden of synonymous variants in 703 genes (two-sided Fisher’s exact test). There is no significant inflation between the case and control cohort.

To this end, we first screened a manually curated list of 703 genes (including known POI-causative genes) implicated in ovarian function (Methods and Supplementary Table 6). Most of these genes were involved in different stages of follicle initiation and development, including gonadogenesis, oogenesis, folliculogenesis, oocyte maturation and ovulation. To minimize bias, we removed genes with a mean coverage of informative reads in the coding region <30× in either the cases or the controls, and a final set of 646 genes was included in further association analyses (Supplementary Table 7). Furthermore, the burden tests of synonymous variants showed that there was no significant inflation of background rate between the case and control cohorts (Extended Data Fig. 6).

**Extended Data Fig. 7:**
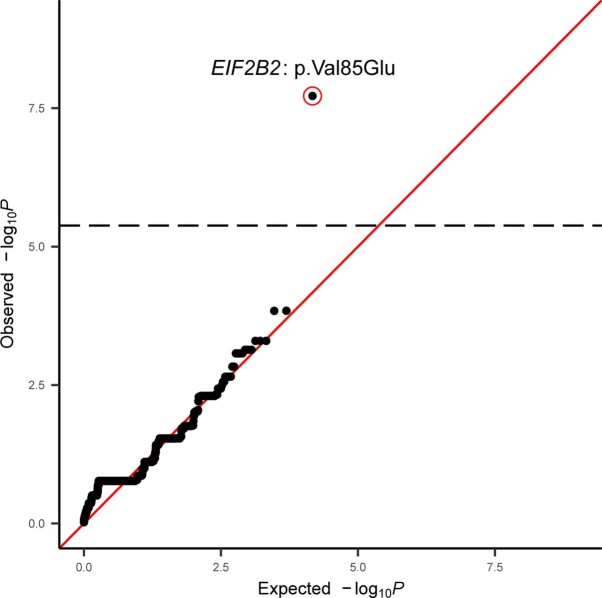
Association analyses of rare coding variants between cases with POI and in-house controls. The quantile-quantile plot comparing observed versus expected *P* values for each rare coding variants in 703 genes (cases n = 1,030, controls n = 5,000, one-sided Fisher’s exact test). The dashed line represents the Bonferroni-corrected P <0.05 threshold. *EIF2B2* p.Val85Glu is the only variant that significantly associated with POI.

In the coding model (exonic and splice region variants), all qualifying variants that met specific criteria (Methods) were subjected to association analyses using one-sided Fisher’s exact tests, which identified 41,046 variants across 639 genes in the control cohort and 11,981 variants across 628 genes in the case cohort. As a result, *EIF2B2* p.Val85Glu was the only variant that stood out in variant-level association tests (Extended Data Fig. 7).

**Fig. 3:**
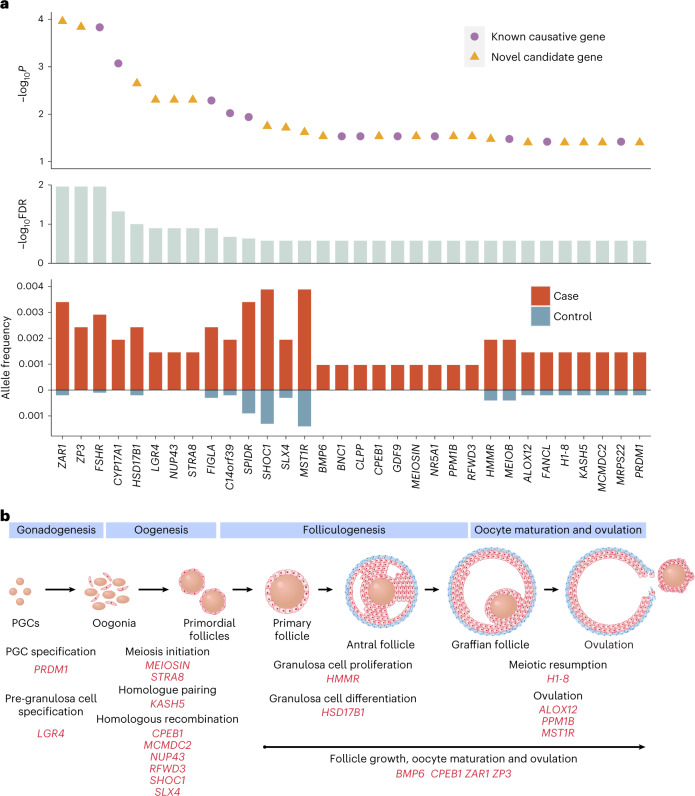
Discovery of novel causative genes through large case–control association analysis of POI. **a**, LoF variants in 32 genes were enriched in cases with POI when compared with controls (cases *n* = 1,030; controls *n* = 5,000). Genes with FDR < 0.3 are shown. The upper graph shows *P* values for difference in the prevalence of LoF variants between cases and control individuals generated by one-sided Fisher’s exact tests; middle graph shows FDR; lower graph displays the allele frequency of LoF variants in each gene. **b**, Overview of 20 novel genes with LoF variants significantly enriched in POI. The upper graph is a schematic representation of the ovary development process, categorized into four stages: gonadogenesis, oogenesis, folliculogenesis and oocyte maturation and ovulation. The lower graph depicts the physiological roles and molecular mechanisms throughout ovary development of 20 significantly enriched genes. *LGR4* and *PRDM1* are involved in gonadogenesis; *KASH5*, *CPEB1*, *MCMDC2*, *MEIOSIN*, *NUP43*, *RFWD3*, *SHOC1*, *SLX4* and *STRA8* are involved in various meiotic processes; and *ALOX12*, *BMP6*, *CPEB1*, *H1-8*, *HMMR*, *HSD17B1*, *MST1R*, *PPM1B*, *ZAR1* and *ZP3* are involved in follicle development, oocyte maturation and ovulation. Genes may be engaged in multiple processes.

A gene-based collapsing approach was then used to identify novel candidate genes. In brief, we identified rare (MAF < 0.001) likely LoF variants, or likely damaging missense (D-mis) variants, and we then aggregated the qualifying alleles into genes and tested for differences between cases and controls using one-sided Fisher’s exact test. The LoF model included 1,439 variants across 433 genes identified in the total cohort (cases and controls). After multiple test correction using the Benjamini–Hochberg method, a false discovery rate (FDR) of 0.3 was set as the threshold. Finally, 32 genes passed the threshold for significantly higher burden of LoF alleles in cases (Fig. 3a). Among these, 20 genes were not previously implicated in patients with POI. In particular, *ZAR1* exhibited the greatest enrichment, followed closely by *ZP3*, both of which had an FDR of 1.1% (Fig. 3a).

**Extended Data Fig. 8:**
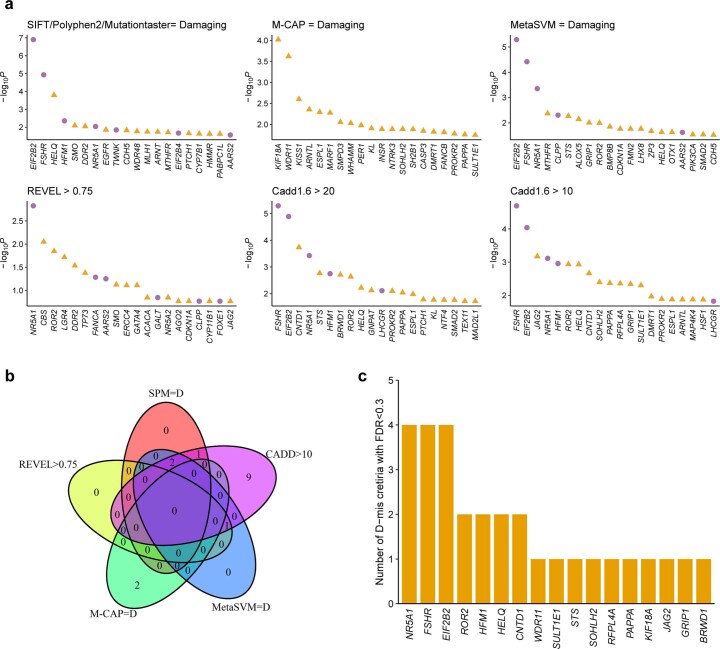
Association analysis for damaging missense variants between POI and controls. **a**, *P* value for the difference in the burden of damaging missense variants evaluated by different algorithms in individual genes (one-sided Fisher’s exact test). The top 20 genes are shown. **b**, Venn diagrams showing the intersection of significantly enriched genes classified according to different D-mis criteria. **c**, The number of D-mis criteria that each gene met.

Additionally, in the D-mis model, we used multiple algorithms to identify genes with significantly more detrimental missense variants in cases than in controls (Methods). Only three POI genes (*NR5A1*, *FSHR* and *EIF2B2*) were enriched for more variants predicted to be damaging by at least four criteria with FDR < 0.3 (Extended Data Fig. 8). However, all three genes are well known to cause POI. The absence of additional pathogenic genes in this analysis may be due to difficulties in evaluating the pathogenicity of missense variants.

**Extended Data Fig. 9:**
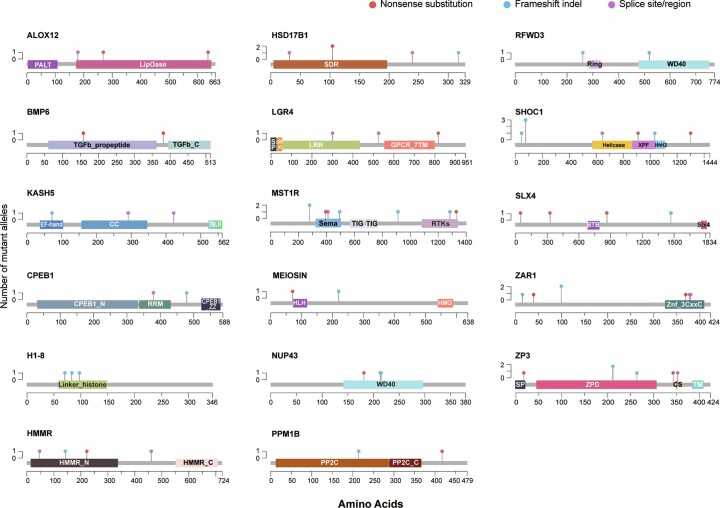
Locations of LoF variants and their affected domains in the proteins encoded by 17 novel significantly enriched genes. Variants in relation to critical functional domain or motifs are depicted. Different types of variants are shown as solid circles and distinguished by color. The x axis represents the number of amino acid residues and the y axis represents the number of variants. Abbreviations of protein domains: BTB, Broad-Complex, Tramtrack and Bric a brac; CC, Coiled-coil domain; CS, Cleavage site; GPCR_7TM, GPCR, rhodopsin-like, 7 transmembrane domain; HhH2, Helix-harpin-helix DNA-binding domain; HLH, Helix-loop-helix DNA-binding domain; HMG, High-mobility group domain; LipOase, Lipoxygenase domain; LR, Luminal region; LRR, Leucine-rich repeat domain; PALT, Polycystin-1, Lipoxygenase, Alpha-Toxin domain; PP2C, Protein serine/threonine phosphatase 2C, catalytic domain; Ring, Ring finger domain; RRM, RNA recognition motifs; RTKs, Receptor tyrosine kinase domain; SDR, Short-chain dehydrogenase/reductase; SP, Signal peptide; TGFb: Transforming growth factor beta like domain; TIG: Immunoglobulin-like fold domain; TM, Transmembrane domain; WD40, WD40-repeat-containing domain; XPF, XPF-like central domain; Znf_3CxxC, Zinc fingers, 3CxxC-type; ZPD, Zona pellucida domain.

Taken together, 20 novel candidate genes were identified by gene-based collapsing analysis in the LoF model. We further investigated their functions (Fig. 3b) and evaluated the pathogenicity of each variant according to ACMG guidelines. The distribution of LoF variants across different locations and the key functional domains involved are shown in Extended Data Fig. 9. The function of novel candidate genes and the LoF variants’ potential deleterious effects are detailed in Supplementary Table 8.

### *ZAR1* and *ZP3* had the strongest associations with POI

*ZAR1* had the highest probability of association (*P* = 1.1 × 10^−4^), largely driven by the presence of seven LoF alleles in cases but only two in controls (Fig. 3a,b). *ZAR1* was one of the first maternal effect genes identified in mammals^15^. It is abundantly expressed in human oocytes in growing and pre-ovulatory follicles, where it performs multiple roles in folliculogenesis, oocyte maturation and embryonic development^16^. *Zar1*-null female mice have a normal number of follicles until meiotic maturation and zygotic genome activation are blocked^17^. In contrast, *Zar1*^*−/−*^ zebrafish exhibit early arrest of oogenesis resulting from aberrant de-repression of the *zona pellucida* (*ZP*) gene mRNA translation^16^. However, despite extensive research in various animal models, no deleterious variant of *ZAR1* has been reported thus far in women with infertility. In the present study, six patients were identified to carry *ZAR1* variants, including one with compound heterozygous and five with heterozygous. All of the LoF variants were predicted to disrupt the conserved C-terminal ZNF domain, through which ZAR1 interacts with ZP or other target mRNAs, thereby impeding its ability to regulate target gene translation.

*ZP3* had the second most significant association with POI (*P* = 1.5 × 10^−4^), with five LoF alleles in cases and none in controls. *ZP3* exerts pleiotropic effects on ovarian development because it is a critical component of the zona pellucida starting from the primordial follicle stage^18^. Interestingly, only missense variants or in-frame deletions in *ZP3* have been reported in patients with defects in oocyte maturation^19,20^. However, these LoF mutations, which were identified in the current POI study, tend to induce more severe protein defects, possibly due to loss of the conserved ZP domain and transmembrane domain.

Moreover, *ZAR1* and *ZP3* appearing as the strongest signals in enrichment analysis illustrates the crucial role that genes involved in follicle development and oocyte maturation play in POI. Among the 20 novel candidate genes, *HMMR*^21,22^, *HSD17B1* (ref. ^23^) and *BMP6* (refs. ^24,25^) have been implicated in follicle development through their regulation of granulosa cell division or steroidogenesis, whereas *H1-8* (ref. ^26^), *PPM1B*^27,28^, *ALOX12* (ref. ^29^) and *MST1R*^30^ are involved in oocyte maturation or ovulation through various mechanisms, such as lipid metabolism and inflammatory response.

### Enriched gonadal development and meiosis-related genes in POI

**Fig. 4:**
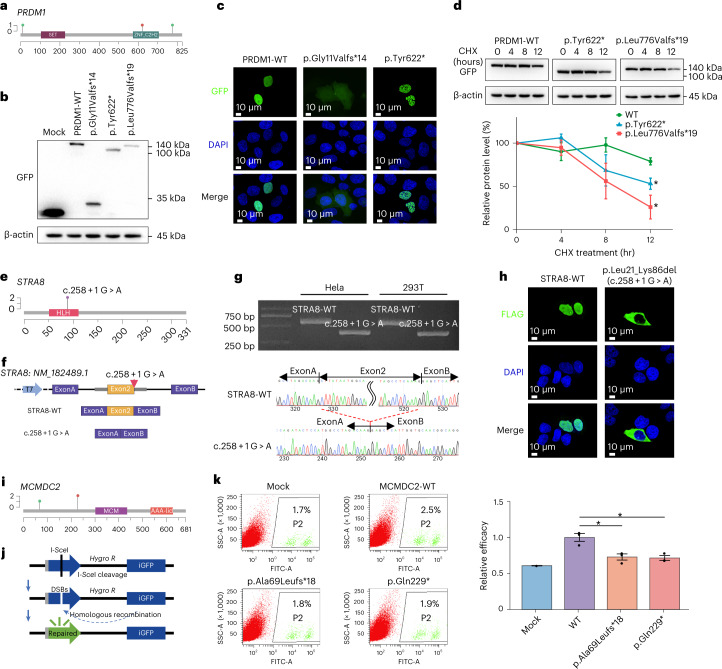
Experimental validation of LoF variants identified in *PRDM1*, *STRA8* and *MCMDC2*. **a**, Map of LoF variant locations relative to essential functional domains in *PRDM1*. **b**, Western blots of transiently expressed WT, p.Gly11Valfs*14, p.Tyr622* and p.Leu776Valfs*19 mutants of GFP-tagged PRDM1 in HEK293 cells. Data are representative of two independent experiments. **c**, Representative fluorescence microscopy images of transiently expressed WT, p.Gly11Valfs*14 and p.Tyr622* mutants of GFP-tagged PRDM1 in HeLa cells. Data are representative of three independent experiments. **d**, Top: western blots of WT, p.Tyr622* and p.Leu776Valfs*19 mutants of GFP-tagged PRDM1 at 0 hours, 4 hours, 8 hours and 12 hours in HEK293 cells from CHX chase assays. Bottom: quantification of PRDM1 protein levels normalized to β-actin. **e**, Map of c.258 + 1 G > A location relative to essential functional domain in *STRA8*. **f**, Schematic representation of mini-gene assay strategy and splicing mode of STRA8-WT and c.258 + 1 G > A. **g**, Agarose gel electrophoresis and Sanger sequencing chromatograms of cDNA after transfection of STRA8-WT or c.258 + 1 G > A into HeLa and 293T cells. Data are representative of two independent experiments in two cell lines. **h**, Representative fluorescence images of transiently expressed WT and p.Leu21_Lys86del (c.258 + 1 G > A) mutant of FLAG-tagged STRA8 in HeLa cells. Data are representative of three independent experiments. **i**, Map of LoF variant locations relative to essential functional domains in *MCMDC2*. **j**, Schematic diagram illustrating the principles of HR assays (Methods). **k**, Left: representative flow cytometry profiles measuring the proportion of cells with DNA repair by HR (GFP^+^ cells) after transfection with WT, p.Ala69Leufs*18 and p.Gln229* mutants of MCMDC2 among HEK293 cells with a GFP-based I-SceI-cleavable reporter. **d**,**k**, Representative data from *n* = 3 biological replicates. Data are shown as means ± s.e.m. Two-sided *t*-test was used to determine significance. The asterisk refers to *P* < 0.05 compared with WT. SET, SET domain; ZNF_C2H2, zinc fingers, C2H2 type; HLH, helix-loop-helix DNA-binding domain; MCM, MCM P-loop containing nucleoside triphosphate hydrolase domain; AAA-lid, AAA-lid domain found in MCM proteins. Source data

The establishment of ovarian reserve relies on the well-orchestrated development of female gonads and meiosis with HR repair proceeding correctly. *PRDM1* encodes a crucial transcriptional regulator required for specification and migration of primordial germ cells (PGCs)^31,32^. Three heterozygous LoF variants were identified in *PRDM1* (Fig. 4a), and functional experiments were performed to validate their pathogenicity. Western blotting revealed that p.Gly11Valfs*14 and p.Tyr622* resulted in truncated proteins, whereas p.Leu776Valfs*19 resulted in substantially reduced expression (Fig. 4b). Furthermore, in contrast with the uniform nuclear distribution observed in the wild-type (WT) GFP fusion protein, the p.Gly11Valfs*14 variant was expressed in both the nucleus and cytoplasm, more similar to the pEGFP empty vector group, whereas p.Tyr622* was concentrated in large, distinct puncta, possibly attributable to protein self-aggregation resulting from abolished DNA binding (Fig. 4c). In addition, p.Tyr622* and p.Leu776Valfs*19 exhibited reduced PRDM1 protein stability after cycloheximide (CHX) treatment compared with the WT (Fig. 4d). By contrast with the PGC-associated candidate, the novel candidate gene *LGR4* was shown to participate in gonadal development by regulating pre-granulosa cell specialization^33^.

In addition, meiotic genes were strikingly enriched (9/20, 45%) among these candidates. *STRA8* is well known to be responsible for triggering meiotic entry and transcriptional activation of meiotic prophase-related genes^34,35^. One homozygous splice site variant c.258 + 1 G > A was found in the present POI cohort (Fig. 4e), whereas no biallelic LoF variant was identified in our in-house controls, and no homozygous LoF variant was found in any public population databases. Mini-gene assays verified that *STRA8* c.258 + 1 G > A caused exon2 skipping, leading to a 66-amino acid in-frame deletion (p.Leu21_Lys86del) in the highly conserved nuclear localization and DNA-binding region of *STRA8* (Fig. 4f–g)^36^. Further immunofluorescence staining revealed that this *STRA8* mutant was restricted to the cytoplasm (Fig. 4h), which was consistent with observations in N-terminal deleted *Stra8*^Δ121/Δ121^ mice^35^, indicating impairment of its transcriptional activation functions and abolished capacity to initiate meiosis.

*MCMDC2*, which promotes homologue alignment and crossover formation during meiosis prophase I, is preferentially expressed in the gonads^37^. One homozygous (p.Gln229*) and one heterozygous (p.Ala69Leufs*18) variant were identified, both of which eliminate the critical MCM and AAA-lid domains^37^ (Fig. 4i). Further GFP-based HR repair efficiency assays (Methods) verified that variants exhibited an HR efficiency 20% that of WT (Fig. 4j–k), potentially impeding HR progression during oocyte meiosis.

Other meiotic genes among the 20 candidates, including *CPEB1* (refs. ^38,39^), *KASH5* (ref. ^40^), *MEIOSIN*^41^, *NUP43* (ref. ^42^), *RFWD3* (refs. ^43,44^), *SHOC1* (ref. ^45^) and *SLX4* (ref. ^46^), play multiple roles during meiotic initiation, homologous pairing, synapsis and HR repair. Animal models with defects in these genes presented infertility, atrophic ovaries and meiotic arrest at different stages, confirming their essential roles involved in meiosis prophase I in the maintenance of ovarian reserve.

The functional annotations of these 20 genes suggest their considerable relevance to POI, with all 20 having a significantly higher burden of LoF variants that could alter gene expression or biological function, as exemplified by experimental validation of *PRDM1*, *STRA8* and *MCMDC2* (Fig. 4). These collective data strongly suggest the likelihood that these 20 genes may be previously unrecognized POI-causative genes.

### Stepwise increases in genetic contribution of POI

**Fig. 5:**
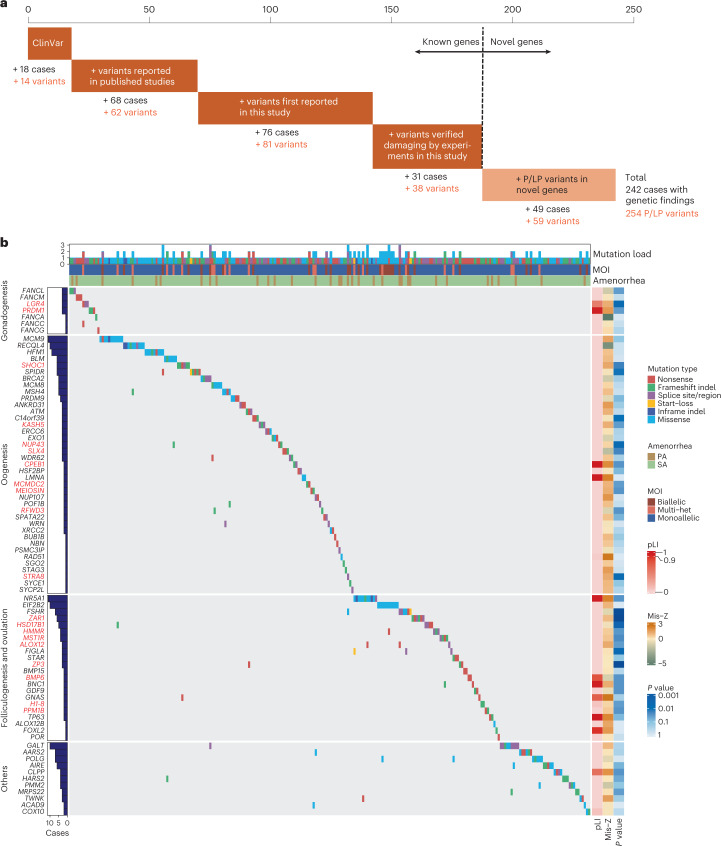
Landscape of P/LP variants identified in known causative genes and novel POI-associated genes. **a**, Contributions of each step, based on varying degrees of evidence, in the analytical pipeline in identifying P/LP variant in 1,030 patients with POI. In total, 193 patients had P/LP variants in known genes, and an additional 49 patients had P/LP variants in novel causative genes. **b**, Integrated matrix of P/LP variants and the 242 patients with detected variants. Rows are genes grouped by ovarian development stages, and columns are patients with POI. The upper panels show patient mutation load, phenotype information and mode of inheritance (MOI). The left panel shows the number of patients carrying P/LP variants in each gene. The right panels show the pLI, Mis-*Z* and raw *P* values of genes using one-sided Fisher’s exact tests.

In the present study, we followed a pipeline through different lines of evidence to identify and validate pathogenic variants and increased the scope of understanding about the contribution of genetic defects in the pathogenesis of POI (Fig. 5a). Known causative genes accounted for 18.7% (193/1030) of cases, of which 86 cases were attributable to 76 variants previously described in ClinVar or published studies, and an additional 107 cases were explained by 119 variants that were reported as damaging in this work. Furthermore, the discovery of novel POI-associated genes introduced 59 P/LP variants, all of which were LoF variants, found in 61 cases. Among these patients carrying variants of novel POI-associated genes, 49 had no P/LP variants in known genes, yielding an additional contribution of 4.8%. Consequently, the rate of contribution to POI by genetic variations reached 23.5% (242/1030) in this study.

We generated an integrated matrix of pathogenic variants identified in known causative genes and novel POI-associated genes (Fig. 5b and Supplementary Table 9). Among the different functional gene groups, no significant clusters were observed in modes of inheritance or mutation load. Genes involved in gonadogenesis tended to have high probability of LoF intolerance (pLI) scores, corresponding to the identification of these genes with LoF variants in the cases. Missense *Z*-score (Mis-*Z*) did not appear to be associated with any functional gene groups. Six genes had Mis-*Z* exceeding 1.96; however, no P/LP missense variants were observed in *TP63* or *CPEB1*. Missense variants were the predominant mutation type in *EIF2B2* and *POLG* although with relatively low *Z*-scores. Overall, both LoF and missense variants substantially contributed to POI, and pLI could serve as a rough guide for prioritizing new POI genes, whereas Mis-*Z* were relatively uninformative. It should be noted that the high heterogeneity of POI makes detailed, gene-specific analyses indispensable.

### Gene sets associated with POI

**Extended Data Fig. 10:**
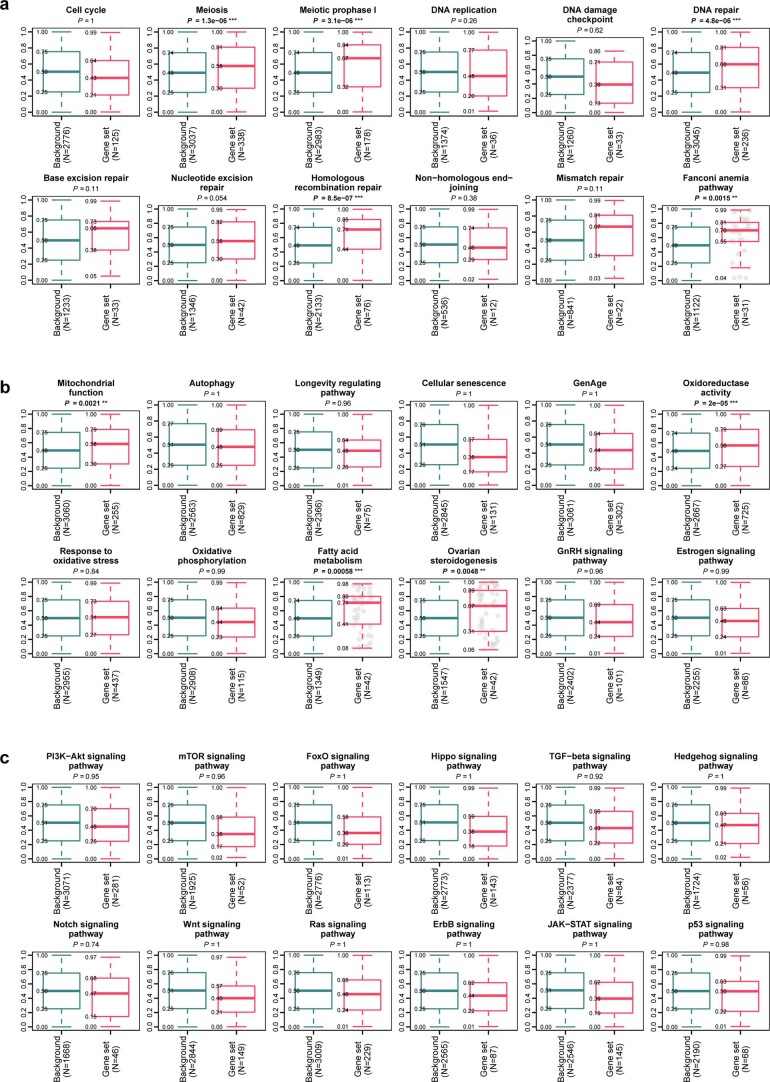
Gene set analysis. Gene-level associations of rare LoF variants between 1,030 cases with POI and 5,000 in-house control individuals are calculated from one-sided Fisher’s exact test, and then we used one-sided Wilcoxon rank sum test between each gene set and comparison set to conduct gene set analysis and generate set-level *P* values. * *P* < 0.05, ** *P* < 0.01, *** *P* < 0.001. The ordinate shows rank percentiles (1 = highest, 0 = lowest) for gene-level associations within background genes and those genes linked to each gene set. Labels indicate minimum, 25^th^ percentile, median, 75^th^ percentile and maximum. N in x-axis represents the number of genes used in association tests. **a**, Gene sets regarding cellular process, DNA replication and DNA repair. **b**, Gene sets regarding metabolism, aging and endocrinology. **c**, Gene sets regarding signal transduction.

Even if a single gene-level association does not reach statistical significance under the constraints of limited sample size, as is the case with most known POI genes, the cumulation of non-significant genes but having mild trends may still be informative when prioritizing candidate genes as relevant or increasing susceptibility to POI. A combination of gene signals in gene-set-level analysis can provide insight into the pathogenesis of POI or give clues toward the detection of novel genes. Therefore, we performed some preliminary analyses in 36 gene sets potentially relevant to ovarian function (Methods and Supplementary Table 10), and the results are shown in Extended Data Fig. 10. Genes implicated in meiosis and DNA repair had significant set-level associations (*P* = 1.3 × 10^−6^ and *P* = 4.8 × 10^−6^, respectively), revealing their likely non-trivial roles on POI^47–49^. Among the subgroups of DNA repair, nucleotide excision repair (*P* = 0.054) also emerged as potentially relevant pathway, in addition to the well-established HR repair^50^ (*P* = 8.5 × 10^−7^) and Fanconi anemia (FA) pathways^51^ (*P* = 1.5 × 10^−3^). This large-scale human genetic study also confirmed the roles of oxidoreductase activity^52^ (*P* = 2.0 × 10^−5^) and fatty acid metabolism (*P* = 5.8 × 10^−4^) in POI. Moreover, the finding that LoF variants in Mendelian mitochondrial disorder-related genes (*P* = 2.1 × 10^−3^) are also significantly enriched in POI provides a link between mitochondrial and ovarian dysfunction. This discovery indicates the possible benefits of monitoring ovarian function in patients with mitochondrial disease.

## Discussion

Here we report, to our knowledge, the largest-scale WES study of POI conducted to date, and we provide a detailed characterization of its genetic landscape. To ensure the reliability of our data, we excluded patients with aberrant karyotypes or other acquired etiologies, adopted ACMG standards to classify variant pathogenicity and used uniformly processed data from a large control population to minimize false positives from possible subpopulations. Upon integrating the 59 known causative genes with 20 novel POI-associated genes identified in the present study, a total of 254 P/LP variants were ultimately identified in 23.5% of the patients with POI. Additionally, P/LP variants in top-ranked genes were detected in less than 1.2% of cases, highlighting its remarkably high genetic heterogeneity.

The substantial contribution of genetic variants to POI in this cohort should prompt reconsideration of routine mutation screening in diagnosed patients, which is not currently recommended in POI management guidelines due to the presumed rarity of monogenic causes^53^. The findings of this large cohort investigation indicate that at least 18~23% of patients have genetic abnormalities, thus supporting implementation of routine clinical WES in POI. Such screening could facilitate determination of patient etiologies and guide genetic counseling for the proband’s female relatives. Genetic testing is particularly beneficial for patients with SA, because their development of POI could be a gradual process, spanning occult (normal FSH level with reduced fecundity), biochemical (elevated FSH level with regular menses) and overt (irregular menses) stages^1^. For women at high risk, alerted by genetic mutations, modest alteration on ovarian reserve indicates the need for timely fertility guidance, including family planning, fertility preservation or assisted reproduction technology.

The link between clinical manifestation of PA or SA and genotype has long presented a challenge to understanding the basis of POI. Previous genetic studies suggest that patients with PA are more likely to have biallelic defects in a single gene^54^, which is confirmed by phenotypic analyses in this report (Fig. 2f). In addition, previous studies have inferred that patients with SA are likely to have multiple defects across various genes coupled with environmental interactions. However, we found a higher frequency of multi-het variants in PA (2.5%) than in SA (1.2%), suggesting that oligogenic models^55^ should be considered in the etiology of POI, regardless of the age at onset of amenorrhea. Comprehensively, our findings support the likelihood that the accumulation of multiple genetic defects may result in a more severe phenotype. Additionally, because *FSHR* mutations are notably more prevalent in PA, we reviewed the clinical characteristics of the carriers. Interestingly, two patients with PA had small ovaries with needle-like follicles, age-appropriate anti-Mullerian hormone (AMH) levels and could be categorized as resistant ovary syndrome^56^. Given that patients with resistant ovary syndrome may achieve pregnancy via in vitro maturation or in vitro activation, genetic screening in patients with PA may have great clinical importance for individualized therapeutic interventions.

The novel candidate genes identified in this study are involved in several processes that were previously unrecognized to play a role in human POI, such as PGC specification, meiosis initiation and maternal mRNA metabolism. Pathogenic variants of *ZAR1*, a maternal effect gene, were reported in human patients with POI and have a remarkably high prevalence. Identification of POI-associated variants in *ZP3* broadens the phenotypic spectrum of this gene beyond its currently understood role in empty follicle syndrome^20,57^. The discovery of variants in *PRDM1* demonstrates that the pathophysiology of POI may begin as early as PGC specification. Although *STRA8* and *MEIOSIN* are well known for their roles in initiating meiosis in animal models, our findings highlight their critical roles in human ovarian disease. Additionally, mutations in *SHOC1* and *KASH5* have been reported in men with non-obstructive azoospermia^58^, and this report described their variants in women with POI, implying that these meiotic genes are shared genetic determinants in both female and male human gametogenesis.

Due to the limitations of WES, systematic analyses of non-coding regions, copy number variations and structural variations in POI were not conducted here. Beyond these technical limitations, our sample size in this cohort still lacks sufficient statistical power to detect much rarer associated genes due to the high genetic heterogeneity of POI. One indication of this heterogeneity is the lack of significance in a large proportion of known POI-causative genes. In addition, the stringent criteria in ACMG guidelines resulted in exclusion of a proportion of missense variants from classification as causative in the absence of experimental data. Despite the larger size of this study compared to previous efforts, the genetic contribution to POI reported here is likely to represent an underestimation.

In summary, this study provides a detailed characterization of pathogenic variants in POI, broadening the scope of known POI-associated genes, to depict the genetic landscape of this disease. Larger cohort size, parent-proband trio sequencing, advanced genomic technologies and international collaborative studies are critical for overcoming the limitations of this study to further determine the genetic etiologies of POI. In addition, mapping the genetic landscape of individuals with differences in ovarian function, such as decreased ovarian reserve, early menopause and POI, may aid in understanding the common genetic factors in reproductive aging.

## Methods

### Participants

#### Patients

All procedures involving patients in this study were approved by the institutional review board of Reproductive Medicine of Shandong University (approval number 2014IRB52). A total of 1,030 female patients with POI from the Reproductive Hospital Affiliated to Shandong University were recruited in the present study. Written informed consent was obtained from each participant. The diagnosis criteria for POI were oligo/amenorrhea for at least 4 months and elevated serum FSH levels >25 IU L^−1^ on two occasions (>4 weeks apart) before 40 years of age^53^. Each patient underwent chromosomal analysis, pelvic ultrasound and a thorough examination of the patientʼs medical history. Individuals with etiologies such as chromosomal abnormalities, histories of ovarian surgery or chemotherapy, radiotherapy or autoimmunity disorders were excluded. Patients with POI were further categorized to PA and SA for phenotypic analysis. PA is defined as the absence of menarche before age 16 years, and SA refers to a spontaneous menstrual cycle at least once. The age at recruitment of patients in this study ranged from 16 years to 40 years. All patients self-reported as females, and ultrasound and karyotype confirmed their sex.

#### Controls

For association tests, we applied WES data of 5,000 unrelated individuals (including 2,739 females and 2,261 males, age range 16−83 years) from the HuaBiao project^10^ as a human population control in this study. This project was approved by the Human Ethics Committee of Fudan University. All participants provided written consent for the extraction and storage of their DNA samples and future usage of their DNA data for research. Their sex and age are self-reported.

All the participants voluntarily involved in the study, and no compensation was provided.

### WES and variant calling

Exome sequencing was performed on genomic DNAs extracted from peripheral blood samples of all 1,030 patients with POI, captured with AIExome V1-CNV (iGeneTech) and sequenced on NovaSeq platforms (Illumina) with 150-bp paired-end reads. Sequence reads were aligned to the human reference genome GRCh37/hg19 using Burrows–Wheeler Aligner (BWA 0.7.17) MEM^59^. Removal of duplicate reads and variant calling of single-nucleotide variants and small indels were used (Genome Analysis Toolkit (GATK 4.1.8.1))^60^. Annotation of variants was used (Ensembl Variant Effect Predictor (VEP 100))^61^ with the RefSeq database. Variants with a genotype call rate >95%, MAF > 1% and LD-pruned based on maximum r_2_ = 0.2 (parameters: -indep-pairwise 50 5 0.2) were selected for identity-by-descent analyses using PLINK 1.9 (ref. ^62^). All participants were confirmed to be unrelated to each other, with the PI-HAT value of less than 0.185.

### Interpretation of variants

The pathogenicity of variants in this study was manually determined according to ACMG guidelines^11^, and the details of criteria we used are listed in Supplementary Table 3. Those variants were classified as P/LP in the ClinVar database with criteria provided by multiple submitters, and no conflicts or those that were reviewed by an expert panel were also considered^63^. Only variants classified as P or LP were reported here, and all of them were confirmed by Sanger sequencing.

### Gene list determination

#### Known causative genes

Human genes that were considered known POI causal genes were all previously identified deleterious variants in patients with syndromic or isolated POI, and their causative association was evaluated by functional verification in animal models or in vitro studies or by observing co-segregation with POI in large families or co-occurrence in multiple unrelated patients. We generated the list of known POI genes by searching the PubMed and OMIM databases for articles published up to December 2021, using terms related to genes (for example, ‘gene’, ‘genetic’, ‘mutation’ or ‘variant’) in conjunction with terms related to POI (for example, ‘ovarian insufficiency’, ‘ovarian failure’, ‘ovarian dysgenesis’, ‘ovarian aging’, ‘ovarian dysfunction’, ‘gonadal failure’, ‘gonadal dysgenesis’, ‘reproductive dysfunction’ or ‘hypogonadism’). The roles of genes in POI etiology were then carefully evaluated for each unique search result. In total, a list of 95 known causative genes was compiled as well, and the associated phenotypes references for each known POI gene are listed in Supplementary Table 2.

#### Candidate gene list for collapsing analyses

We manually curated a list of 703 genes with established associations with ovarian function as follows: (1) genes whose mutations had been implicated in the development of isolated or syndromic reproductive diseases caused by abnormal ovarian function, such as POI and oocyte maturation defect; (2) genes whose disruptions in mouse models yielded impairment of ovarian function according to Mouse Genome Informatics (MGI; http://www.informatics.jax.org/) and the International Mouse Phenotyping Consortium (https://www.mousephenotype.org/) database; and (3) genes with functional verification by in vitro studies or in other animal models (for example, zebrafish and flies). Gene lists compiled by refs. ^6,64^ were referenced.

#### Gene sets

Meiosis and meiotic prophase I gene sets were curated based on the functional association per MGI and literature review. The DNA repair gene set and its subsets were curated from the updated Human DNA Repair Genes^65^ (https://www.mdanderson.org/documents/Labs/Wood-Laboratory/human-dna-repair-genes.html) and the REPAIRtoire dabatase^66^, whereas genes in the FA set containing 22 FA genes and nine FA-associated genes were compiled by ref. ^51^ and ref. ^67^. The mitochondrial function gene set consisting of 255 nuclear genes reported to cause mitochondrial disease was curated from ref. ^68^, ref. ^69^ and ref. ^70^. The autophagy gene set was curated from the Autophagy Database^71^ and the Human Autophagy Database^72^. The GenAge set includes 307 genes associated with human aging in the GenAge database^73^. Gene sets of oxidoreductase activity (GO:0016705) and response to oxidative stress (GO:0006979) were curated based on the Gene Ontology database (http://geneontology.org/).

Other gene sets were curated based on the Kyoto Encyclopedia of Genes and Genomes (KEGG) database (https://www.genome.jp/kegg/), including pathways of cell cycle (PATH:ko04110), DNA replication (PATH:ko03030), longevity regulating (PATH:ko04211), cellular senescence (PATH:ko04218), oxidative phosphorylation (PATH:ko00190), fatty acid metabolism (PATH:hsa01212), ovarian steroidogenesis (PATH:ko04913), GnRH signaling (PATH:ko04912), estrogen signaling (PATH:ko04915), PI3K-Akt signaling (PATH:ko04151), mTOR signaling (PATH:ko04150), FoxO signaling (PATH:ko04068), Hippo signaling (PATH:ko04390), TGF-beta signaling (PATH:ko04350), Hedgehog signaling (PATH:ko04340), Notch signaling (PATH:ko04330), Wnt signaling (PATH:ko04310), RAS signaling (PATH:ko04014), ErbB signaling (PATH:ko04012), JAK-STAT signaling (PATH:ko04630) and p53 signaling (PATH:ko04115). All genes in gene sets are listed in Supplementary Table 10.

### Statistical analysis

The case cohort and the control cohort used in this study were captured with the same exome enrichment kit, and the same standardized bioinformatics pipeline was applied. Cases and controls showed similar sequencing metrics (Supplementary Table 5). To minimize bias, only the genes with mean coverages of coding regions greater than 30× both in the two cohorts were included in our association analyses (Supplementary Table 7).

Qualifying coding variants were defined based on the following criteria: (1) exonic or splice region; (2) mean read depth (DP) > 10; (3) alternative allele read frequency ≥25%; (4) mean quality by depth (QD) < 20; (5) mean phred quality (QUAL) < 30; and (6) mean genotype phred quality (GQ) < 20. We used a MAF cutoff of 0.001 either in the global population or the East Asian population from the gnomAD database (http://gnomad.broadinstitute.org/). Synonymous variants, most of which are presumed benign, were usually used to determine whether there is a preferential inflation of background variation. To further confirm the lack of preferential inflation of background variation, we assessed the tallies of rare qualifying synonymous variants per individual and burden tests of each synonymous variant. As a result, both of them did not show a significant difference between the case and control cohorts (Extended Data Fig. 6).

For the gene-level collapsing analysis, we ran two models: the LoF and the D-mis model. The LoF model included only LoF variants (start–loss, canonical splice site, frameshift and nonsense) removing at least the last 2% of amino acids. For the D-mis model, multiple algorithms were used to predict deleteriousness of missense variants, and we set six criteria to define D-mis: (1) SIFT < 0.05, Polyphen2 > 0.15 and MutationTaster predicted as deleterious; (2) predicted as ‘possibly pathogenic’ by M-CAP; (3) predicted as ‘deleterious’ by MetaSVM; (4) REVEL > 0.75; (5) CADD > 20; and (6) CADD > 10. D-mis determined by different criteria were separately analyzed in parallel, and their results were compared. The number of alleles in the cases was compared with those in controls across 646 genes using one-sided Fisher’s exact tests.

For gene set enrichment analysis, we first constructed a comparison set consisting of the 50 nearest neighbors in the genome of each gene within the gene set. The genes in the gene set and the corresponding matched genes were combined and were applied the same quality control criteria as above. Gene-level associations of LoF variants were calculated for each gene between cases and in-house controls. The gene-level *P* values were then ranked, and a one-sided Wilcoxon rank-sum test was performed in each gene set to assess whether the genes in the gene set ranked significantly higher than the comparison genes.

### Phasing of two heterozygous variants

Multiple approaches were applied to confirm the phase of two variants detected in one gene of a single individual in circumstances where parental DNA samples were unavailable. Variants located within the 150-bp region (POI-572: *NR5A1*) were phased using GATK HaplotypeCaller and reviewed manually using Integrative Genomics Viewer software^74^.

For variants ranging in size from 150 bp to ~10 kb pairs (POI-506: *AARS2*; POI-910: *RECQL4*; POI-991: *AARS2*; POI-1151: *EIF2B2*; and POI-1660: *ZAR1*), phasing was determined using TA cloning sequencing. A genomic DNA fragment covering the two heterozygous variants was amplified through LA Taq DNA polymerase (Takara). PCR products were purified with a gel extraction kit (BioTeke) and cloned into T-Vector pMD20 (Takara). The construct products were transformed into *Escherichia coli* competent cells. At least four bacterial colonies were collected and cultured overnight in LB medium containing 100 μg ml^−1^ of ampicillin (Solarbio). Plasmid DNA was isolated and verified by Sanger sequencing. Phasing of the two variants was determined by analyzing whether they occurred in the same or distinct clones. The primers, antibodies and other commercial reagents used in the present study are listed in Supplementary Tables 11 and 12.

For variants with a distance over ~10 kb pairs (POI-169: *NR5A1*; POI-516: *HFM1*; POI-841: *MCM9*; POI-1228: *MCM9*; and POI-1453: *MSH4*), phasing was determined using 10x Genomics as described previously^75^. High-molecular-weight genomic DNA (>50-kb pairs) was extracted using the Magnetic Blood Genomic DNA Kit (TIANGEN) from the peripheral blood. The sample-indexed sequencing libraries were prepared via the GemCode platform (10× Genomics) and sequenced on NovaSeq platforms (Illumina) with 150-bp paired-end reads according to the manufacturerʼs protocol. Average coverage of each sample was around 30×, and the sequence data were 128 Gb.

### Plasmids and mutagenesis

The full-length cDNA of *PRDM1* was purchased and cloned into pEGFP-C1. The mutant *PRDM1* overexpression plasmids were generated by overlap extension PCR. The methods to construct plasmids used in the minigene splicing assay of *STRA8* are described in detail below. To validate the function of exon2 deletion of *STRA8*, the full-length cDNA of *STRA8* was PCR amplified from human transcriptome cDNA and cloned into p3 × FLAG-CMV7.1 as the WT plasmids, and the exon2 deletion mutant *STRA8* overexpression plasmid was generated using overlap extension PCR.

The WT overexpression plasmids of *BLM*, *HFM1*, *MCMDC2*, *MCM8*, *MCM9*, *MSH4* and *RECQL4* cloned in pcDNA3.1-3 × FLAG-C were purchased from YOUBAO Biology. The WT overexpression plasmid *NR5A1* in pENTER was purchased from Vigene Biosciences. All the mutant overexpression plasmids were generated through QuickChange Lightning Site-Directed Mutagenesis Kit (Agilent Technologies) according to the manufacturerʼs protocol.

### Cell culture and plasmid transfection

HEK293 (Procell), 293T (China Center for Type Culture Collection), HeLa (Procell) and CHO (Procell) cell lines used for in vitro experiments in this study were all derived from females. Cells were cultured at 37 °C and 5% CO_2_ and grown in DMEM (Gibco) or Ham’s F-12K medium (Gibco), supplemented with 10% FBS (Gibco) and 1% penicillin–streptomycin (Gibco). When cells reached the appropriate confluence, they were transfected with plasmids using Lipofectamine 3000 (Invitrogen) according to the manufacturerʼs protocol in the absence of antibiotics, and, 6 hours later, the media were replaced with fresh complete DMEM or F12-K culture media containing FBS and penicillin–streptomycin.

### Protein blotting and CHX chase assay

HEK293 cells were transfected with wild or mutant pEGFP-C1-*PRDM1* overexpression plasmids. pEGFP-C1 vector was also transfected as the negative control group. At 48 hours after transfection, cells were harvested and lysed in RIPA lysis buffer (Beyotime) with 1% Protease Inhibitor Cocktail (Cell Signaling Technology). The total protein was quantitated using a BCA protein assay kit (Thermo Fisher Scientific) according to the manufacturerʼs instructions. Total protein (20 μg) of each sample was loaded, separated on an SDS–PAGE gel and transferred to a polyvinylidene fluoride membrane (MilliporeSigma). The membrane was blocked, incubated with GFP antibody (1:10,000 dilution, Abcam) and anti-rabbit secondary antibodies (1:5,000 dilution, Proteintech) and with β-actin antibody (1:5,000 dilution, Proteintech) and anti-mouse secondary antibody (1:5,000 dilution, Proteintech). The membranes were scanned using a Chemidoc MP Imaging System (Bio-Rad). Two independent experiments were carried out.

For the CHX chase assay, CHX (Beyotime) was added to the culture medium 24 hours after transfection at a concentration of 0.01 mM. Upon treating with CHX for 0 hours, 4 hours, 8 hours and 12 hours, cell samples were collected and stored at −80 °C until western blot analysis was performed. For each timepoint, three independent experiments were carried out.

### Minigene splicing assay

A minigene splicing assay was conducted to examine the function of splicing site mutation c.258 + 1 G > A identified in *STRA8*. WT *STRA8* fragment with restriction sites (*Xho*I and *Bam*HI) encompassing 3′ terminal intron1 (220 bp), exon2 (198 bp) and 5′ terminal intron2 (382 bp) was obtained from human genomic DNA through nested PCR amplification. c.258 + 1 G > A located in the donor site of intron2 was introduced by overlap extension PCR. WT or mutant *STRA8* fragment were digested and then ligated into pcMINI vector containing ExonA-IntronA-multiple cloning site-IntronB-ExonB (Bioeagle Biotech Company). The pcMINI-*STRA8* constructs were transfected into HeLa and 293T cell lines, and cells were harvest after 48 hours. Total RNA was extracted using TRIzol reagent (Invitrogen) and then reverse transcribed to cDNA. cDNA was PCR amplified using primers flanking the minigene. PCR products were separated by agarose gel electrophoresis. Each band was gel purified and then sequenced to determine the transcripts of WT and mutant constructs. It is worth noting that this experiment was repeated in two cell lines, HeLa and 293T, with each cell line being transfected once with WT and mutant constructs.

### Immunofluorescence microscopy

To evaluate the effects of variants detected in *PRDM1* and *STRA8* on protein location or expression profiles, immunofluorescence microscopy was conducted according to standard techniques as described previously^76^. In brief, HeLa cells were cultivated on glass coverslips in 12-well plates and transfected with expression plasmids at the appropriate density. At 36 hours after transfection with WT and mutant pEGFP-N1-*PRDM1* constructs, cells were fixed with 4% paraformaldehyde, mounted and stained for nuclei using antifade reagent containing DAPI (Beyotime). After transfection with WT and mutant p3 × FLAG-CMV7.1-*STRA8* constructs, fixation, permeabilization and blocking of non-specific antibody binding (in 1× PBS containing 0.3% Triton X-100 and 10% BSA) were performed. Cells were then stained with FLAG antibody (1:300 dilution, Cell Signaling Technology) and goat anti-rabbit secondary antibody conjugated with Alexa Fluor 488 (1:800 dilution, Invitrogen) before mounting and staining for nuclei. Sealed coverslips were visualized under a confocal microscope (ANDOR Technology), and immunofluorescence pictures were captured by performing *z*-axis scan at 5-μm intervals. Three independent experiments were performed.

### HR repair efficiency assay

To investigate the effects of variants detected in genes implicated in the HR repair pathway (*BLM*, *HFM1*, *MCM8*, *MCM9*, *MCMDC2*, *MSH4* and *RECQL4*), a stable HEK293 cell line carrying a GFP-based I-*Sce*I-cleavable reporter (provided by Fengli Wang from Huazhong University of Science and Technology and Hailong Wang from Capital Normal University) was adopted as previously described^77^. Lentiviral I-SecI expression plasmid was infected into cells to generate double-strand breaks (DSBs). Around 24 hours later, WT or mutant HR gene overexpression plasmids were transfected to cells. pcDNA3.1 vector was also transfected as the negative control group. After culturing for 48 hours, cells were harvest for flow cytometry analysis on an LSRFortessa Cell Analyzer (BD Biosciences) to quantitate the number of GFP^+^ cells and total cells. If the DSBs were repaired by the means of HR, the GFP would be expressed; thus, HR repair efficiency was evaluated by the percentage of GFP^+^ cells in total cells. Three independent experiments were conducted, with a minimum of 30,000 cells counted for each group.

### Luciferase assays

Luciferase assays were used to determine the effect of variants identified in *NR5A1* on transcriptional activity. Chinese hamster ovary (CHO) cells were seeded in 24-well plates. The cells were co-transfected with WT or mutant overexpression plasmids (pENTER-*NR5A1*) and pEZX-PG04.1-*CYP19A1* reporter plasmids. Simultaneously, pENTER was transfected as a negative control group. The cell culture medium was collected 48 hours after transfection, and the luciferase activity was determined using the Secrete-Pair dual luminescence kit (GeneCopoeia) according to the manufacturerʼs protocol. The luminescent activity of GLuc and SEAP were measured by the Enspire luminometer reader (PerkinElmer). Results were normalized to the activity of SEAP luciferase. Three individual experiments were carried out.

### Reporting summary

Further information on research design is available in the Nature Portfolio Reporting Summary linked to this article.

## Online content

Any methods, additional references, Nature Portfolio reporting summaries, source data, extended data, supplementary information, acknowledgements, peer review information; details of author contributions and competing interests; and statements of data and code availability are available at 10.1038/s41591-022-02194-3.

## Supplementary information

Reporting Summary

Supplementary Tables 1–12 Supplementary Table 1: Clinical characteristics of 1,030 recruited patients with POI. Supplementary Table 2: List of 95 known POI-causative genes. Supplementary Table 3: Criteria used in this study for classifying pathogenic variants based on ACMG standards and guidelines. Supplementary Table 4: Clinical presentations related to 15 genes identified as having pathogenic or likely pathogenic variants in at least five cases. Supplementary Table 5: Summary sequencing statistics for the POI and control cohorts. Supplementary Table 6: List of 703 genes related to ovarian function. Supplementary Table 7: Summary coverage statistics of coding region of 703 genes for the POI cohort and the control cohort. Supplementary Table 8: Gene function of the 20 novel POI-associated genes and the presumed fucntion of identified LoF variants. Supplementary Table 9: Information of pathogenic or likely pathogenic variants in 242 cases with POI. Supplementary Table 10: Classification of 36 gene sets analyzed in the present study. Supplementary Table 11: Sequence for primers used in the present study. Supplementary Table 12: Cell lines, antibodies and commercial reagents used in the present study.

## Data Availability

The raw sequencing data of 1,030 patients with POI reported in this study have been deposited in the Genome Sequence Archive (GSA) in the National Genomics Data Center, China National Center for Bioinformation/Beijing Institute of Genomics, Chinese Academy of Sciences, under accession number HRA003245 (project: PRJCA012479), which can be accessed at https://ngdc.cncb.ac.cn/gsa-human/. These data are available under restricted access, as individual genomic sequencing data are protected owing to patient privacy and Regulations on the Management of Human Genetics Resources of China. The raw data can be requested via the GSA-Human System and can be authorized for downloading by the Data Access Committee for research and non-commercial use only. Detailed guidance on data access requests can be found in the repository’s document (https://ngdc.cncb.ac.cn/gsa-human/document/GSA-Human_Request_Guide_for_Users_us.pdf). Accession requests are typically responded to within 2 weeks. The processed genotype dataset in VCF format (including the position, reference allele, mutated allele, allele frequencies and qualities of all variants) is open-accessed via the National Omics Data Encyclopedia and can be freely and publicly downloaded under accession number OEP003709. Variants of the control cohort used in this study were generated by the HuaBiao project and can be obtained from https://www.biosino.org/wepd/. The databases used in analyses are all publicly available and can be obtained from the following links: ClinVar: https://www.ncbi.nlm.nih.gov/clinvar; Human DNA Repair Genes: https://www.mdanderson.org/documents/Labs/Wood-Laboratory/human-dna-repair-genes.html; REPAIRtoire: https://repairtoire.genesilico.pl; Autophagy Database: http://tp-apg.genes.nig.ac.jp/autophagy; Human Autophagy Database: http://www.autophagy.lu; Human Ageing Genomic Resources (GenAge): http://genomics.senescence.info; Gene Ontology: http://geneontology.org; and Kyoto Encyclopedia of Genes and Genomes (KEGG): https://www.genome.jp/kegg. Source data are provided with this paper.

## Source data

Source Data Fig. 4 Unprocessed western blots and statistical source data.
Source Data Extended Data Fig. 2 Statistical source data.

## Code availability

Our in-house scripts are available at https://github.com/ShuyanTang/POI1030.
