# Supplementary material for: Landscape of pathogenic mutations in premature ovarian insufficiency
Source: Nat Med. 2023 Feb 2;29(2):483–92. doi: 10.1038/s41591-022-02194-3 (PMC9941050; doi:10.1038/s41591-022-02194-3)
Supplement: Supplementary file 1 — Reporting Summary [file 41591_2022_2194_MOESM1_ESM.pdf]

nature portfolio

Corresponding author(s): Zi-Jiang Chen, Li Jin, Yingying Qin,Feng Zhang

Last updated by author(s): Dec 14, 2022

Reporting Summary

Nature Portfolio wishes to improve the reproducibility of the work that we publish. This form provides structure for consistency and transparency in reporting. For further information on Nature Portfolio policies, see our Editorial Policies and the Editorial Policy Checklist.

Statistics

For all statistical analyses, confirm that the following items are present in the figure legend, table legend, main text, or Methods section.

n/a	Confirmed	
☐	☒	The exact sample size (*n*) for each experimental group/condition, given as a discrete number and unit of measurement
☐	☒	A statement on whether measurements were taken from distinct samples or whether the same sample was measured repeatedly
☐	☒	The statistical test(s) used AND whether they are one- or two-sided<br>*Only common tests should be described solely by name; describe more complex techniques in the Methods section.*
☐	☒	A description of all covariates tested
☐	☒	A description of any assumptions or corrections, such as tests of normality and adjustment for multiple comparisons
☐	☒	A full description of the statistical parameters including central tendency (e.g. means) or other basic estimates (e.g. regression coefficient) AND variation (e.g. standard deviation) or associated estimates of uncertainty (e.g. confidence intervals)
☐	☒	For null hypothesis testing, the test statistic (e.g. *F*, *t*, *r*) with confidence intervals, effect sizes, degrees of freedom and *P* value noted<br>*Give P values as exact values whenever suitable.*
☒	☐	For Bayesian analysis, information on the choice of priors and Markov chain Monte Carlo settings
☒	☐	For hierarchical and complex designs, identification of the appropriate level for tests and full reporting of outcomes
☒	☐	Estimates of effect sizes (e.g. Cohen's *d*, Pearson's *r*), indicating how they were calculated

Our web collection on statistics for biologists contains articles on many of the points above.

Software and code

Policy information about availability of computer code

Data collection	Exome sequencing was performed on genomic DNAs extracted from peripheral-blood samples of all 1,030 female patients with POI, captured with AIExome Enrichment Kit V1 (iGeneTech, Beijing, China) and sequenced on Illumina NovaSeq platforms (Illumina, San Diego, CA) with 150-bp paired-end reads. Genomic vcf files of the control cohort from 5,000 individuals (including 2,739 females and 2,261 males) were obtained from the HuaBiao project, which were captured with the same exome enrichment kit and were applied the same quality control criteria as cases.
Data analysis	Our in-house scripts are available at https://github.com/ShuyanTang/POI1030.<br>The softwares used in this study include:<br>BWA 0.7.17: http://bio-bwa.sourceforge.net/;<br>CADD 1.6: https://cadd.gs.washington.edu/<br>FACS Diva v6.1.3: https://www.bdbiosciences.com/en-us/products/software/instrument-software/bd-facsdiva-software<br>GATK 4.1.8.1: https://gatk.broadinstitute.org/;<br>MetaSVM 1.0: https://github.com/jjh0925/metaSVM<br>MutationTaster 2021: https://www.mutationtaster.org/<br>PLINK 1.9: https://www.cog-genomics.org/plink/1.9/;<br>Polyphen 2: http://genetics.bwh.harvard.edu/pph2/<br>SIFT 2: http://github.com/jdtournier/mrtrix3<br>VEP 100: https://grch37.ensembl.org/info/docs/tools/vep/;

For manuscripts utilizing custom algorithms or software that are central to the research but not yet described in published literature, software must be made available to editors and reviewers. We strongly encourage code deposition in a community repository (e.g. GitHub). See the Nature Portfolio guidelines for submitting code & software for further information.

Data

Policy information about availability of data

All manuscripts must include a data availability statement. This statement should provide the following information, where applicable:

- Accession codes, unique identifiers, or web links for publicly available datasets
- A description of any restrictions on data availability
- For clinical datasets or third party data, please ensure that the statement adheres to our policy

The raw sequencing data of 1,030 POI cases reported in this study have been deposited in the Genome Sequence Archive (GSA) under the accession number HRA003245 (Project: PRJCA012479) that are publicly accessible at https://ngdc.cncb.ac.cn/gsa-human/. These data are available under restricted access, as individual genomic sequencing data are protected due to patient privacy and Regulations on the Management of Human Genetics Resources of China. The raw data can be requested via GSA-Human System, and can be authorized for downloading by the Data Access Committee only for research and non-commercial use. The detailed guidance on data access requests can be found in the repository's document (https://ngdc.cncb.ac.cn/gsa-human/document/GSA-Human_Request_Guide_for_Users_us.pdf). Accession requests are typically responded to within two weeks. The processed genotype dataset in vcf format (including the position, reference allele, mutated allele, allele frequencies, and qualities of all variants) is open accessed via the National Omics Data Encyclopedia and can be freely and publicly downloaded under the accession number OEP003709 (https://www.biosino.org/node/project/detail/OEP003709).
Variants of the control cohort used as in this study were generated by the HuaBiao project and can be obtained from https://www.biosino.org/wepd/.
The databases used in analyses are all public available and can be obtained from the following links:
Clinvar: https://www.ncbi.nlm.nih.gov/clinvar;
Human DNA Repair Genes: https://www.mdanderson.org/documents/Labs/Wood-Laboratory/human-dna-repair-genes.html;
REPAIRtoire: https://repairtoire.genesilico.pl;
Autophagy Database: http://tp-apg.genes.nig.ac.jp/autophagy;
Human Autophagy Database: http://www.autophagy.lu;
Human Ageing Genomic Resources (GenAge): http://genomics.senescence.info;
Gene Ontology: http://geneontology.org;
Kyoto Encyclopedia of Genes and Genomes (KEGG): https://www.genome.jp/kegg

Field-specific reporting

Please select the one below that is the best fit for your research. If you are not sure, read the appropriate sections before making your selection.

☒ Life sciences ☐ Behavioural & social sciences ☐ Ecological, evolutionary & environmental sciences

For a reference copy of the document with all sections, see nature.com/documents/nr-reporting-summary-flat.pdf

Life sciences study design

All studies must disclose on these points even when the disclosure is negative.

Sample size

Our study includes genomic and phenotype information for a retrospective cohort of 1,030 patients diagnosed with POI and without chromosome abnormalities and known non-genetic causes. No sample size-calculations were performed since we recruited as many samples as we can under the low incidence of POI, and this is the largest exome sequencing study of POI to date. Moreover, this sample size provides enough power to detect some genetic associations of POI that have not yet been identified before. Negative results may need to be revisited for detecting much rarer associated genes when larger cohorts become available in the future.

Controls are consisted of 5,000 individuals from the HuaBiao project. Sex was not considered in our study design per se, because POI is specifically a female reproductive disorder and all 1,030 patients in the current cohort are female. 5,000 anonymous individuals (including 2,739 females and 2,261 males) from the HuaBiao project serve as population genome controls in this study to investigate the potential enrichment of rare pathogenic variants in the patient cohort by compapring with the allele frequencies in Chinese populations.

Data exclusions

Patients with chromosome abnormalities and known non-genetic causes of POI (including autoimmunity diseases, ovarian surgery, chemotherapy, or radiotherapy) were excluded.

Replication

According to our finding that the top-ranked gene was only detected in less than 1.2% of cases revealing the remarkably high genetic heterogeneity, it is unlikely to replicate in a smaller cohort as this is the largest exome sequencing study of POI to date. Hence no replication study were performed.

Pathogenic or likely pathogenic variants reported in this study have been confirmed by Sanger sequencing. For laboratory experiments, at least two independent experiments were performed, which are indicated in the figure legends and the Methods section.

Randomization

Randomization was not applicable as this is an observational study .

Blinding

Blinding of the samples was not applied because no intervention was conducted in this study.

Reporting for specific materials, systems and methods

We require information from authors about some types of materials, experimental systems and methods used in many studies. Here, indicate whether each material, system or method listed is relevant to your study. If you are not sure if a list item applies to your research, read the appropriate section before selecting a response.

Materials & experimental systems

n/a	Involved in the study
☐	☒ Antibodies
☐	☒ Eukaryotic cell lines
☒	☐ Palaeontology and archaeology
☒	☐ Animals and other organisms
☐	☒ Human research participants
☒	☐ Clinical data
☒	☐ Dual use research of concern

Methods

n/a	Involved in the study
☒	☐ ChIP-seq
☐	☒ Flow cytometry
☒	☐ MRI-based neuroimaging

Antibodies

Antibodies used

anti-GFP (Abcam, ab183734; 1:10000)
anti-Beta Actin (Proteintech, 66009-1-Ig; 1:5000)
anti-FLAG-tag (Cell Signaling, 14793; 1:300)

Goat anti-rabbit IgG (H+L) Alexa Fluor 488 (Invitrogen, A-11006; 1:800)
HRP-conjugated Affinipure Goat Anti-Rabbit IgG(H+L) (Proteintech, SA00001-2; 1:5000)
HRP-conjugated Affinipure Goat Anti-Mouse IgG(H+L) (Proteintech, SA00001-1; 1:5000)

Validation

anti-GFP (Abcam, ab183734): The manufacturer verified the antibody in human cell line through western blot (https://www.abcam.com/gfp-antibody-epr14104-ab183734.html).
anti-Beta Actin (Proteintech, 66009-1-Ig): The manufacturer verified the antibody in human cell line through western blot (https://www.ptgcn.com/products/Pan-Actin-Antibody-66009-1-Ig.htm).
anti-FLAG-tag (Cell Signaling Technology, 14793): The manufacturer verified the antibody in human cell line through Immunofluorescence (https://www.cellsignal.com/products/primary-antibodies/dykddddk-tag-d6w5b-rabbit-mab-binds-to-same-epitope-as-sigma-s-anti-flag-m2-antibody/14793).

Eukaryotic cell lines

Policy information about cell lines

Cell line source(s)

HEK293 (catalog number CL-0001), HeLa (catalog number CL-0101), and CHO (catalog number CL-0061) were obtained from Procell (Wuhan, CN). 293T (catalog number GDC0187) was obtained from China Center for Type Culture Collection (Wuhan, CN).

Authentication

None of the cell lines used were authenticated.

Mycoplasma contamination

All cell lines tested negative for mycoplasma contamination.

Commonly misidentified lines
(See ICLAC register)

No commonly misidentified cell lines were used in this study.

Human research participants

Policy information about studies involving human research participants

Population characteristics

POI female patients diagnosed as oligo/amenorrhea for at least four months and elevated serum follicle stimulating hormone (FSH) levels > 25 IU/L on two occasions (>4 weeks apart) before 40 years old. Each POI case underwent chromosomal analysis and a thorough examination of the patient's medical history. Subjects with etiologies such as chromosomal abnormalities, histories of ovarian surgery or chemotherapy, radiotherapy, or autoimmunity disorders were carefully excluded.

The control cohort consists of unrelated and self-reported healthy participants. All samples were recruited from the Han Chinese population. The self-reported age of the participants ranged from 16 to 83 years. 2,739 self reported as females and 2,261 self reported as males.

Recruitment

POI patients were enrolled from the Reproductive Hospital Affiliated to Shandong University with written informed consent. As for patients, there might be information bias about the self-reported histories of menstruation and diseases, medicine or treatments affecting ovarian function. The bias of menstrual history would affect the classification of subgroups referring to primary and secondary amenorrhea. The recruitment of patients with diseases, medicine or treatments that adversely affect ovarian function could underestimate the genetic contribution in the etiologies of idiopathic POI.

The control cohort was recruited by the HuaBiao project from the Han Chinese population, who self reported as healthy. There are possibilities that a small number of potential POI patients exist in the control cohort, who did not recognize menses change or did not evaluate ovarian function by hormone test, as well as some did not at the age of disease onset.

	Considering the prevalence of POI in general population is rare (<1%), the bias resulting from potential patients should be slight.
Ethics oversight	All study procedures involving patients were approved by the Institutional Review Board of Reproductive Medicine of Shandong University. Written informed consent was obtained from each participant.<br>The HuaBiao project was approved by the Human Ethics Committee of Fudan University. All participants provided written consent for the extraction and storage of their DNA samples and future usage of their DNA data for research.

Note that full information on the approval of the study protocol must also be provided in the manuscript.

Flow Cytometry

Plots

Confirm that:

☒ The axis labels state the marker and fluorochrome used (e.g. CD4-FITC).

☒ The axis scales are clearly visible. Include numbers along axes only for bottom left plot of group (a 'group' is an analysis of identical markers).

☒ All plots are contour plots with outliers or pseudocolor plots.

☒ A numerical value for number of cells or percentage (with statistics) is provided.

Methodology

Sample preparation	Treated cells in dishes were digested, washed, and resuspended in 1x PBS.
Instrument	LSR Fortessa Cell Analyzer (BD Biosciences, Franklin Lakes, NJ)
Software	Flow cytometry was collected and initially analyzed using FACS DIva v.6.1.3 and FlowJo v.10.5.0
Cell population abundance	Nearly 30000 cells
Gating strategy	The preliminary FSC/SSC gate distinguished all cells from other impurities, and the second gate distinguished GFP positive cell population from GFP negative population.

☒ Tick this box to confirm that a figure exemplifying the gating strategy is provided in the Supplementary Information.
