# Supplementary material for: Landscape of pathogenic mutations in premature ovarian insufficiency
Source: Nat Med. 2023 Feb 2;29(2):483–92. doi: 10.1038/s41591-022-02194-3 (PMC9941050; doi:10.1038/s41591-022-02194-3)
Supplement: Source Data Fig. 4 — Unprocessed western blots and statistical source data. [file 41591_2022_2194_MOESM3_ESM.pdf]

Uncropped merging images of western blot corresponding to Fig.4b

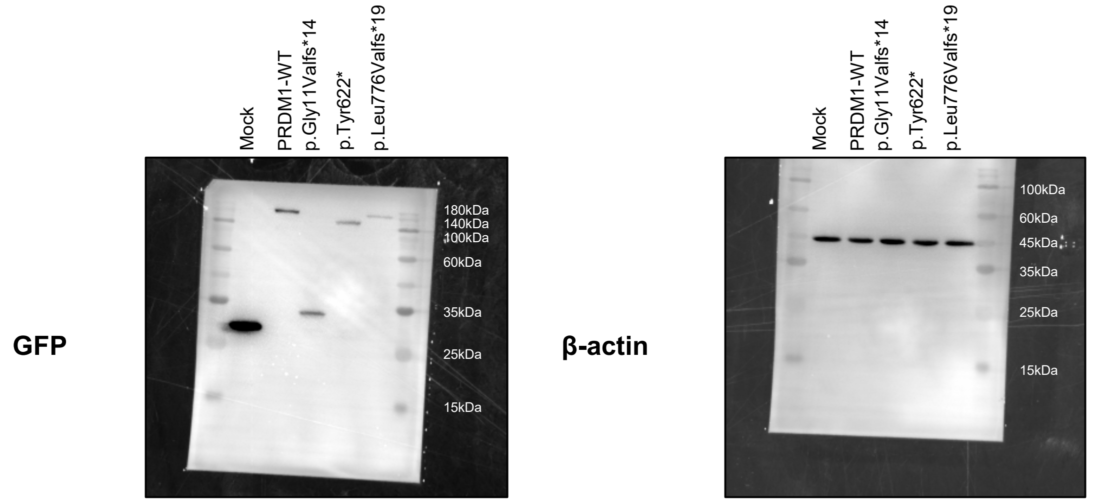

Uncropped merging images of western blot corresponding to Fig.4d

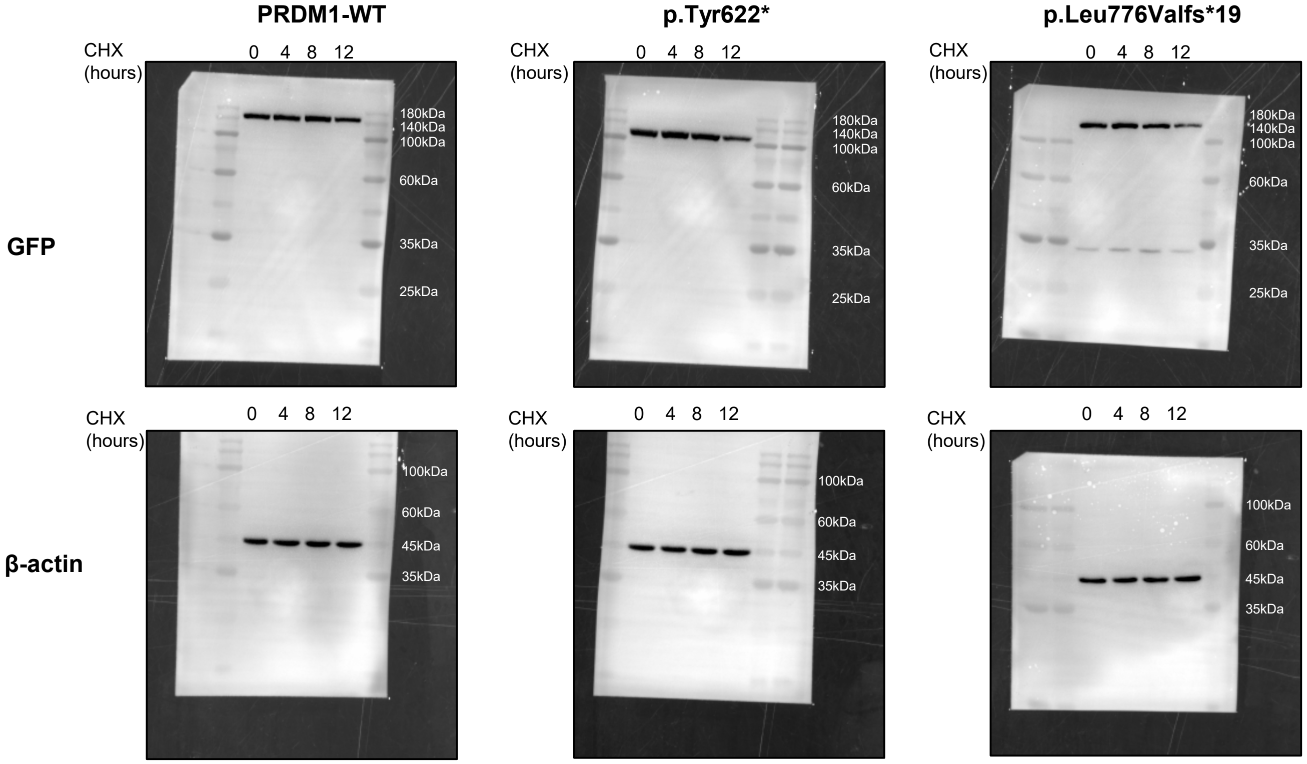

Relative protein level corresponding to Fig.4d

CHX treatment	PRDM1-WT				p.Tyr622*				p.Leu776Valfs*19				*P* value	
	First	Second	Third	mean ± S.E.	First	Second	Third	mean ± S.E.	First	Second	Third	mean ± S.E.	p.Tyr622* *vs.* WT	p.Leu776Valfs*19 *vs.* WT
0 hours	1	1	1	-	1	1	1	-	1	1	1	-	-	-
4 hours	1.0813	0.8959	0.7261	0.9011±0.1026	1.0885	0.9780	1.1189	1.0618±0.0428	1.1061	0.9315	0.8032	0.9469±0.0877	0.222	0.752
8 hours	1.1144	0.8306	0.9902	0.9784±0.0821	0.5175	0.4890	1.0460	0.6842±0.1811	0.9715	0.3657	0.3442	0.5605±0.2056	0.213	0.132
12 hours	0.8381	0.8189	0.7080	0.7884±0.0405	0.4822	0.4491	0.6581	0.5298±0.0649	0.5327	0.1456	0.0998	0.2594±0.1373	0.028	0.021

Gating Strategy corresponding to Fig.4k

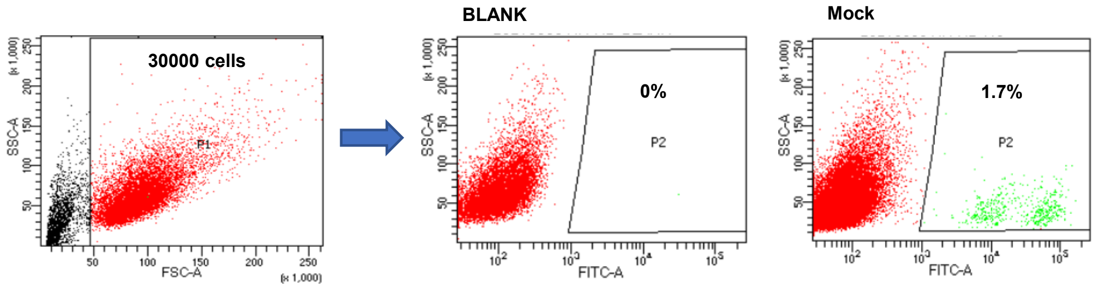

Gating strategy:
The preliminary FSC/SSC gate **(P1)** distinguished all cells from other impurities, the second gate **(P2)** distinguished GFP positive cell populations from GFP negative populations. The proportion of GFP positive cells in Blank group is zero.

Cell population abundance: 30000 cells

Relative GFP positive cells proportion corresponding to Fig.4k

	First	Second	Third	First.after	Second.after	Third.after	Mean.after	S.E.after	*P* value (compared with WT)
Mock	1.0000	1.0000	1.0000	0.6079	0.6079	0.6079	0.6079	0	-
MCMDC2-WT	1.4706	1.7143	1.7500	0.8940	1.0421	1.0639	1.0000	0.0533	-
p.Ala69Leufs*18	1.0588	1.2857	1.2500	0.6437	0.7816	0.7599	0.7284	0.0428	0.017
p.Gln229*	1.1176	1.2857	1.1250	0.6794	0.7816	0.6839	0.7150	0.0333	0.011
